# Breaking Down Polychlorinated Biphenyls and Aryl Chlorides: A Computational Study of Thermal-, Pressure-, and Shear-Induced Decomposition

**DOI:** 10.1021/acs.jpca.4c08086

**Published:** 2025-02-27

**Authors:** L. Pisarova, O. A. Loboda, I. Minami, S. J. Eder

**Affiliations:** † AC2T research GmbH, Viktor-Kaplan-Straße 2/C, 2700 Wiener Neustadt, Austria; ‡ Department of Engineering Sciences and Mathematics, Division of Machine Elements, Luleå University of Technology, SE-97187 Luleå, Sweden; § Institute for Engineering Design and Product Development, TU Wien, Lehárgasse 6 − Objekt 7, 1060 Vienna, Austria

## Abstract

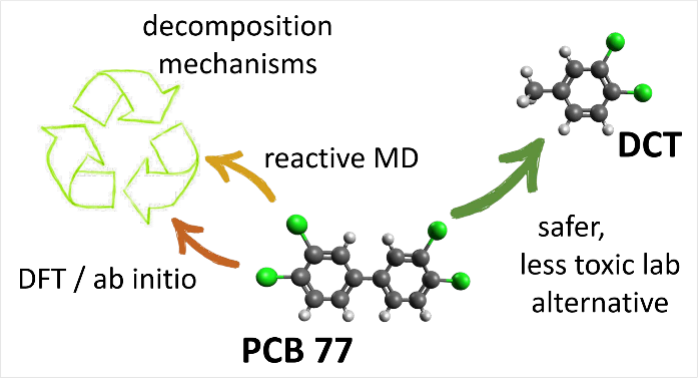

Reactive molecular dynamics (MD) simulations were used to study the decomposition of aryl chlorides, including polychlorinated biphenyls (PCBs), under varying conditions. Using the ReaxFF force field, which models bond breaking and formation, the study focused on PCB 77 (3,3′,4,4′-tetrachlorobiphenyl) and compared it to safer alternatives: 1,2-dichlorobenzene (DCB) and 3,4-dichlorotoluene (DCT). Density functional theory (DFT) calculations validated decomposition pathways and enthalpies of C–Cl bond homolytic cleavage, revealing a multistep radical mechanism. Analysis showed that the decomposition rate and product distribution were sensitive to temperature and Cl-binding positions, emphasizing the complexity of PCB breakdown. Decomposition products were analyzed to understand the efficiency and safety of current remediation processes, such as incineration, which can produce hazardous byproducts like dioxins if poorly managed. The results suggested DCT as a promising candidate for further investigation in laboratory experiments due to its decomposition pathways and relevance to PCB analogues. This study advances knowledge of PCB degradation mechanisms, informing safer, sustainable remediation strategies, and highlighting the risks of pyrolysis-based approaches.

## 1 Introduction

Polychlorinated biphenyls (PCBs) are a group of highly toxic pollutants that have been found to be persistent in various environmental matrices.^1^ They were used extensively in the past for various industrial and commercial purposes^2^ due to their desirable chemical properties, such as thermal and chemical stability, low flammability, and electrical insulation.^3^ However, their widespread use has resulted in significant environmental contamination, and they have been recognized as potent environmental and human health hazards. They are still unintentionally produced as byproducts in dye and paint manufacturing.^4^ PCBs have been shown to cause a variety of negative health effects, including cancer, immune system dysfunction, and reproductive disorders.^5^

The removal of PCBs from the environment is a major challenge, as they are resistant to natural degradation processes.^6^ While some physical and chemical methods have been developed to remove them from contaminated sites, these methods can be expensive, are generally effective and economically viable only for low-concentration levels (ppm = mg/kg) and sensitive to environmental matrices (water, soil), while additionally they may result in the formation of even more toxic byproducts such as dioxins, e.g., via improper incineration.^7^ As a result, there is a need to develop new and efficient methods to decompose PCBs and other stable chlorinated waste, such as aryl-chlorides, in a safe and sustainable manner.

The use of atomistic computer simulations can provide valuable insight into decomposition reaction pathways and help with the selection of suitable safe chemical alternatives used as models in experimental studies for validation purposes. In particular, reactive molecular dynamics (MD) has emerged as a powerful computational tool for studying compound stability due to its ability to simulate even multiple chemical reactions occurring in larger systems over a nanosecond time scale.^8^ The individual influences on the compounds’ stability can be studied in a deconvoluted manner, e.g., shear effects independent of temperature, via temperature control in the constructed system. Shear is the force that causes parallel layers of matter to slide past each other and can cause breaking of chemical bonds, thus aiding decomposition.

Reactive MD enables researchers to investigate the underlying mechanisms of reactions and predict the behavior of complex systems under a range of conditions, such as decomposition of extreme pressure additives leading to protective film formation on ferrous surfaces under purely thermal^9^ or shear conditions,^10^ or the failure of solid lubricants.^11^ Reactive MD is much less computationally expensive compared to, e.g., DFT calculations and can handle larger system sizes (several thousand atoms) and longer simulation durations (several nanoseconds), although DFT is indispensable for the fitting of force fields^12^ or for providing insights into the adsorption on surfaces.^13^ In a recent study, a new force field was presented focusing on organochloride compounds modeling,^14^ illustrating the importance and the sustained interest of the topic. In other recent work, researchers used classical MD to study ligand–receptor interactions in PCBs^15^ and how dioxin-like compounds may disrupt the human endocrine system,^16^ but the main interest here was structure clarification rather than chemical reactions of the molecules.

In this study, we apply reactive MD to provide deep insight into the chemical mechanisms that govern the decomposition of PCBs under elevated temperatures, pressures, and shear. Our primary model compound is PCB 77, a highly toxic, abundantly produced and persistent PCB congener, which is also major PCB congener formed during PO36 pigment manufacturing.^4^ We compare the decomposition properties of PCB 77 to those of dichlorobenzene (DCB) and dichlorotoluene (DCT), two simpler structural analogues to PCB 77 that are less toxic but stable model compounds. We do this in the hope of facilitating safer laboratory experiments while maintaining similar decomposition properties as PCB 77. We investigate the influence of thermal and mechanical conditions on the decomposition of PCB 77, DCB, and DCT by varying the temperature and pressure, and then explore the effect of shear on the decomposition process. Finally, we use other computational methods to aid the interpretation of the observed reaction pathways and decomposition products while utilizing insights from chemical reaction theories. Our study serves as an important initial step before including explicit metallic surfaces with which the molecules can interact chemically. By understanding the fundamental mechanisms that govern PCB decomposition subjected to temperature, pressure, and shear, we hope to develop more efficient and sustainable methods for the elimination of these toxic stable compounds.

## 2 Methods

### 2.1 Studied Chemicals

PCB 77 belongs to the group of tetra-chlorinated PCBs, one of the most abundant homologue groups in PCBs formulations, and has a symmetrical structure from which safer benzene-based model compounds can be derived for experimental studies. Additionally, the ortho positions on the aromatic rings of PCB 77 are not chlorinated, resulting in its planar structure and thus making it one of the highly toxic PCB representatives due to its capability to activate the aryl hydrocarbon receptor (AhR), leading to immunotoxicity, carcinogenicity, and endocrine disruption, among others. Thus, the choice of inclusion of chlorinated benzene-based model compounds in the reactive MD studies was based on the health risk connected to PCB exposure due to toxicity reasons while working with high concentration solutions at a single-digit percentage level (toxic waste) or in neat form during the planned experimental validation study. A second aspect is the prevention of hazardous material generation containing high levels of PCBs during experimental work, thus taking precautions leading not only to human, but also to environmental protection.

**Figure 1:**
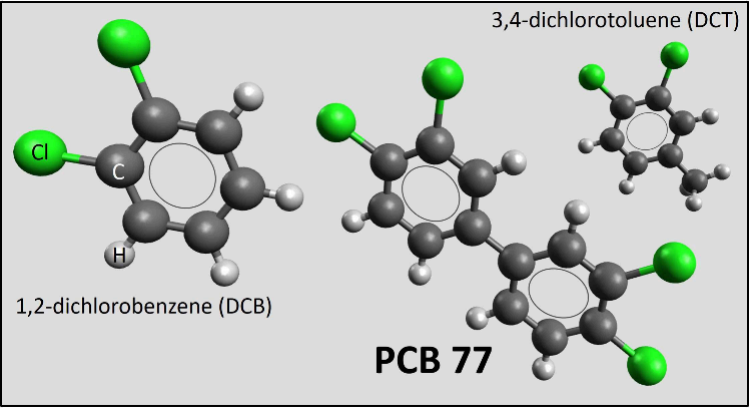
Three studied chemistries.

Hence, the molecules included in this study were PCB 77, as a representative PCB, 1,2-dichlorobenzene (DCB), and 3,4-dichlorotoluene (DCT), see Figure 1.

### 2.2 Simulation and Visualization Details

We used reactive molecular dynamics simulations with the open-source MD code LAMMPS^17,18^ and the reactive force field ReaxFF^19,20^ to investigate the decomposition of chlorinated aromatic compounds under a range of different thermal and mechanical conditions.

#### 2.2.1 Reactive MD: System Setup and Simulation Protocol

**Figure 2:**
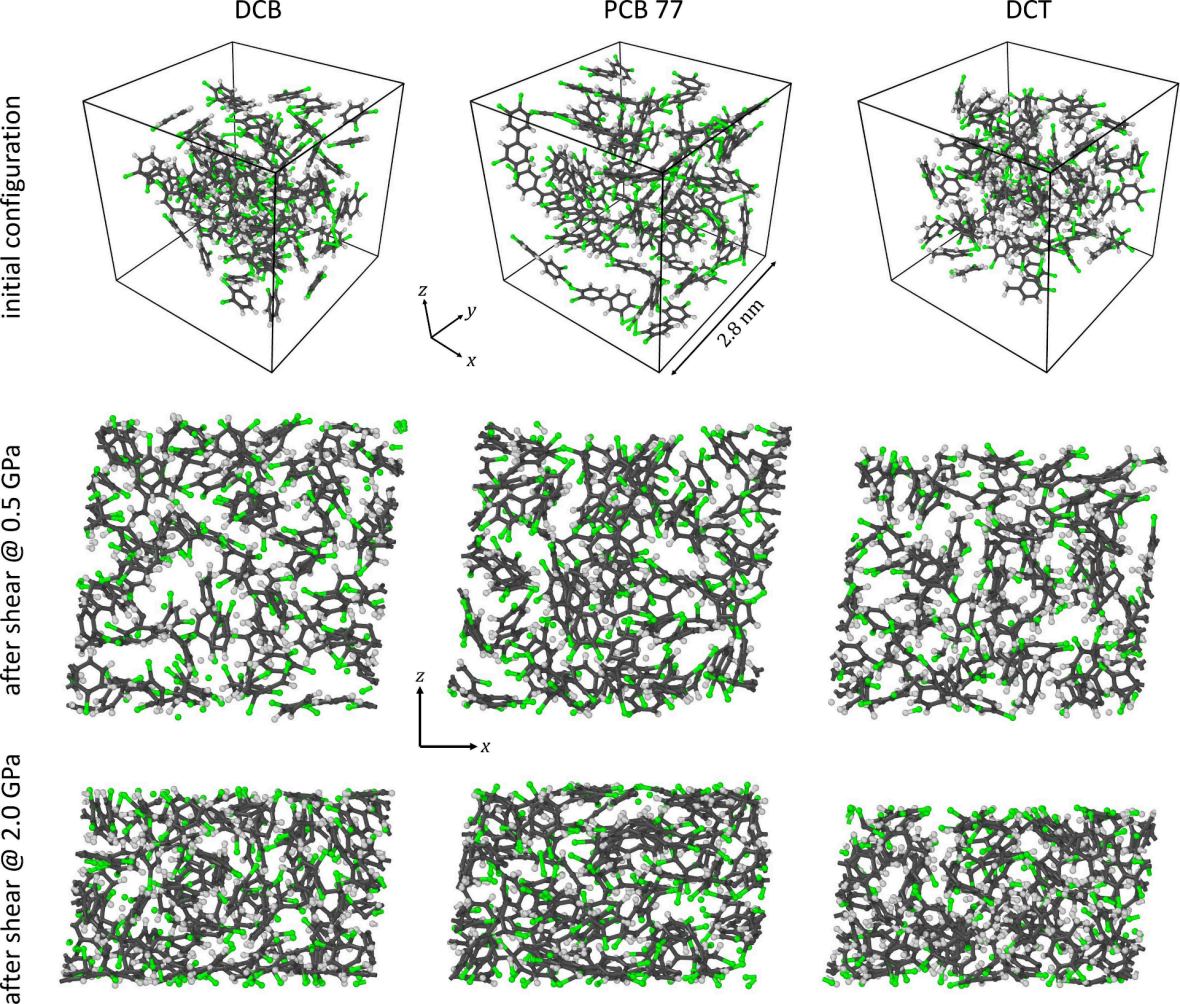
Representative snapshots of the three studied systems. The top row shows 3D representations of the simulation box prior to thermalizing, loading, and shearing, while rows 2 and 3 show side views of the systems after shearing at ∼2100 K at lower 0.5 GPa and higher 2 GPa pressures, respectively. Carbon is gray, hydrogen is white, and chlorine is green.

The reference system was constructed by randomly placing 50 PCB 77 molecules in a cubic simulation box of side length 2.8 nm, see the center of the top row in Figure 2. We attempted to keep the relevant gap thickness (system height) at any particular pressure similar, while studying systems with the three different molecules, to keep the shear strain rate comparable. Thus, not the total number of molecules was kept the same, but rather the total number of atoms, as the molecules vary considerably in size, which would directly affect the gap thickness. This led to the system with 50 molecules of PCB 77 (1100 atoms) being compared to systems of 92 DCB molecules (1104 atoms) and 73 DCT molecules (1095 atoms), see the top row of Figure 2. Care was taken that no molecule initially straddles any simulation box boundary so that any set of boundary conditions could still be applied to the simulation box. The interactions between the atoms and molecules were governed by a ReaxFF force field with parameters taken from ref,^21^ which is able to reliably describe C/H/Cl bonds in the studied aryl chlorides. A Nosé/Hoover thermostat, as implemented in,^22^ with a damping constant of 100 fs was used to control the temperature of the system, and the simulation time step was set to 0.2 fs. For the simulations that featured compression or shear, the SLLOD equations of motion were solved, which are equivalent to Newton’s equations of motion for shear flow^23^ and can in a more general way be used to maintain velocity gradients for all forms of homogeneous flow,^24^ see the center and bottom rows of Figure 2 for representative system snapshots.

Details on molecular stability checks, bond order (BO) threshold determination, thermal degradation studies, and the workflow for compression and shear simulations are provided in the Supporting Information (see Figure S1 for bond order histograms and Figure S2 for the compression-shear workflow).

We then performed shear simulations of 1.2 ns duration at a shear strain rate corresponding to 10^10^ s^–1^ for all three chosen chemicals, selected temperatures of 1500, 1800, 2100, and 2400 K, and applied pressures of 0.5, 1, 2, 3, 4, and 5 GPa. The choice of these, from an experimental angle, quite high temperatures, pressures, and shear strain rate, makes it possible to observe chemical reactions within the tight confinement of the manageable simulation times of around 1 ns, especially when studying stable molecules. Clearly, this sort of molecular activation only serves the purpose of providing the basic kinetic energy requirement so that the chemical reactions may then proceed within a nanosecond time frame. At the same time, where shear dependence is of interest, the computational shear strain rates will typically lie at values several orders of magnitude above those encountered experimentally, although in engineering applications, peak shear strain rates as high as 10^8^ s^–1^ may be encountered in, e.g., in a valve train.^25^ These very high shear strain rates could lead to catastrophic heating of the system in the real world, but temperature within the reactive MD system is carefully controlled via a Nosé/Hoover thermostat, so that temperature, pressure, and shear strain rate are virtually decoupled. In this fashion, it becomes possible to study the influence of any of these parameters individually.

The results obtained from this parametric study described above then served to design a second approach, which attempted to remedy two issues. First, the gap between the high computational shear strain rates and the normally lower experimental ones was somewhat closed by reducing the shear strain rate to an engineering level of 10^8^ s^–1^. Second, we found that the careful mild step-by-step system preconditioning by thermalizing and pressurizing led to substantial loss of the original molecular species even before the shearing could start, which made the shear simulations difficult to compare and kept us from studying the initial decomposition steps within the actual simulation run, as the output from the bond table is registered only after the start of the main simulation (initiation of shearing). Therefore, it was decided to carry out the second parametric study using a “shock-compression and thermalization” step to the target pressure and temperature over a period of only 20 ps (compared to previous system conditioning that lasted up to 3 ns), which was then followed by shear simulations of 1.2 ns duration. This approach was made possible by the knowledge of the correct gap thickness for each system configuration leading to desired pressures, obtained from the stepwise protocol discussed in the Supporting Information.

#### 2.2.2 Visualization and Data Analysis

A central approach to evaluating any MD simulation, reactive or not, is to produce time-dependent visualizations of the system configurations. These can be readily produced using the powerful visualization software OVITO,^26^ which determines bonds based on a simple bond cutoff distance criterion. Care must therefore be taken to define the cutoff radii for the different bond types based on their actual equilibrium bond lengths so that the bonds are correctly identified in OVITO and are widely in agreement with those defined by the ReaxFF bond order criterion. For our visualizations, we used the following cutoff radii: H–H 0.9 Å, C–H 1.2 Å, H–Cl 1.4 Å, C–C 1.6 Å, C–Cl 2.0 Å, and Cl–Cl 2.2 Å corresponding to the respective equilibrium bond lengths. The resulting visualizations can be converted to movies that allow a qualitative tracking of the occurring reactions, see Figure 2 for examples.

Since the pressure is adjusted by setting the correct gap thickness, as described above, the actual pressure that the system saturates to after an extended period of shearing must be logged and compared with the desired value. Variations of ±10% were considered acceptable, but should be kept in mind when comparing, e.g., different chemistries at the “same” selected pressures. The system temperature was also monitored, but this quantity rarely deviates from its target value by any considerable amount due to the effectiveness of the applied thermostat.

**Figure 3:**
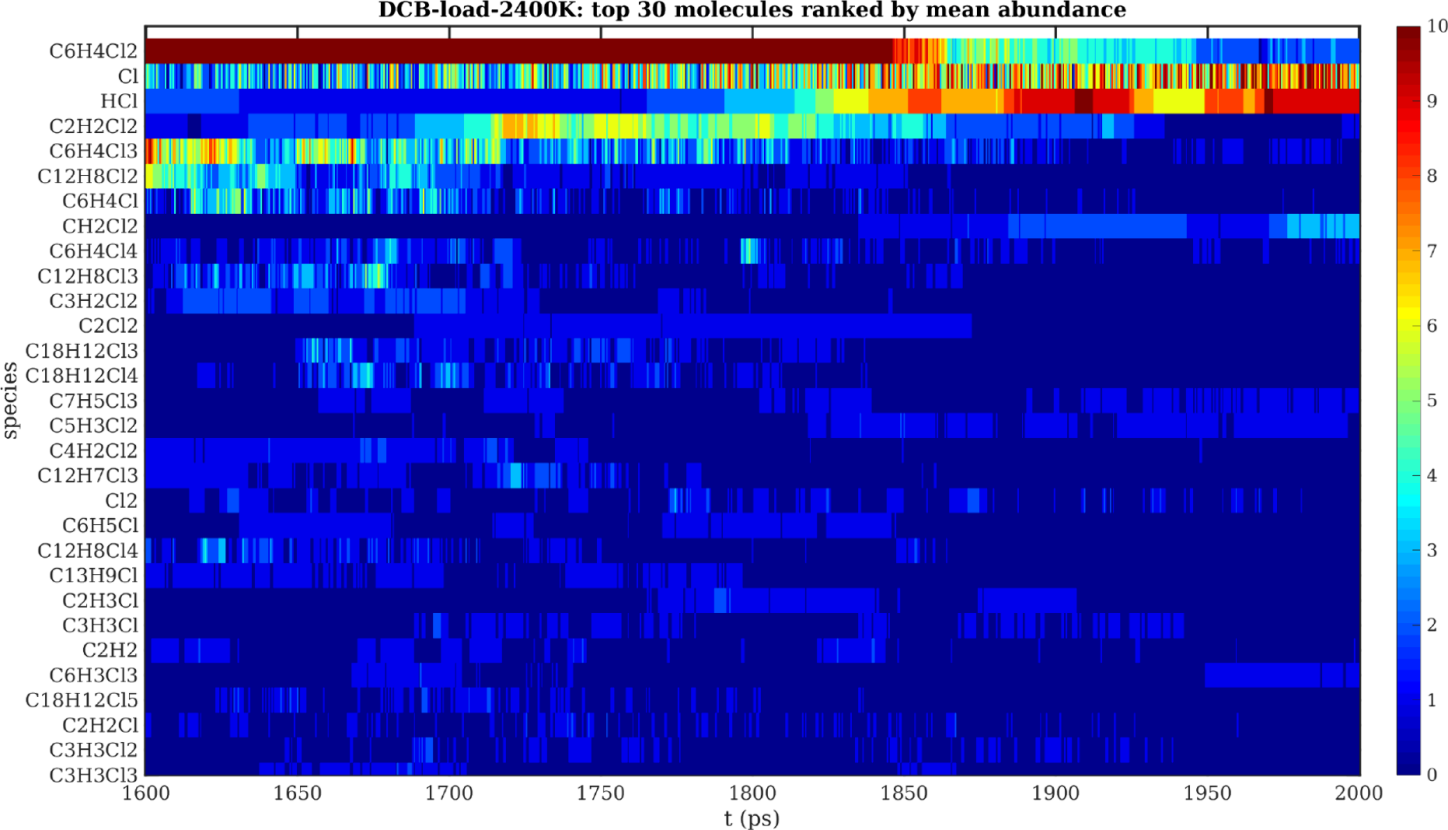
Representative heat map of the 30 chemical species ranked highest in mean abundance while performing a loading simulation with DCB at 2400 K. Note that the color bar was adjusted so that dark red represents only 10 molecules (compared to the 92 initial DCB molecules) so that the less abundant reaction products might be better resolved.

The main part of the analysis, especially in quantitative sense, is based on the output evaluation from the bonds tables provided by LAMMPS and ReaxFF. As mentioned, with an adjusted bond order cutoff at 0.6 value we were able to capture all bond types feasible among combinations of C, Cl, and H, while avoiding unrealistic artifacts. With the input of the bond order threshold, the program mol_fra that is distributed with LAMMPS can translate the bonds table of ReaxFF into a time-dependent list of molecular species in the system presented as chemical formulas (number of atoms of the chemical elements contained in a compound). The resulting output is human-readable text and can be straightforwardly parsed for further analysis. We found it the most instructive to rank the molecular species by minimum, maximum, and mean abundance, as well as by the total time of existence of that species (but without any consideration of its abundance). As hundreds of different species might be registered during the simulation period, especially for high temperature and pressure studies, only the top 30 most abundant molecules (according to any of the above ranking schemes) were selected for further consideration. They were plotted using their chemical formula within a “heat map” color coding style corresponding to their abundance at a respective time step, with time plotted on the abscissa (*x*-axis) and rows listing each of the identified molecular species, see Figure 3. Such maps constitute a valuable resource for identifying the main intermediate and final decomposition products, thus giving first evidence concerning the principal reaction pathways. However, for assessing the stability of the original reactant molecules specifically, such heat maps are less effective due to their upper limit of 10 molecules on the color bar. For such an analysis, e.g., for studying ring openings, 1D plots showing the evolution of some 10 species over time would provide a more practical and accurate representation. From the generated heat maps it was concluded that the first 30 compounds were sufficient to include the most relevant occurring chemical species.

**Figure 4:**
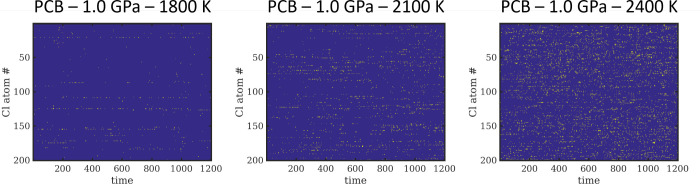
Bond forming and breaking events (yellow) of all 200 Cl atoms in 50 molecules of PCB 77 under shear at 1.0 GPa and three different temperatures as a function of time (in ps).

An in-depth analysis focusing on the chlorine atoms of the simulation can identify all dechlorination and possible chlorination events during the simulation runtime. The output interval of the bonds table (1 ps) constitutes the resolution limit of this analysis approach: any chemistry occurring at lower time scales cannot be properly resolved. A useful way of visualizing this is another type of map with the time on the abscissa and one row corresponding to each of the chlorine atoms in the system, which are uniquely labeled. The rows are then marked in a different color whenever the bonding state of the corresponding chlorine atom has changed with respect to the previous time step (occurrence of bond dissociation or formation). With such a map, one can quickly obtain an overview of the general reactivity of the chlorine atoms in the system and also see if this reactivity might be restricted to certain time periods or to certain groups of molecules. Three examples of such maps for PCB under shear at 1.0 GPa at three different temperatures can be found in Figure 4.

**Figure 5:**
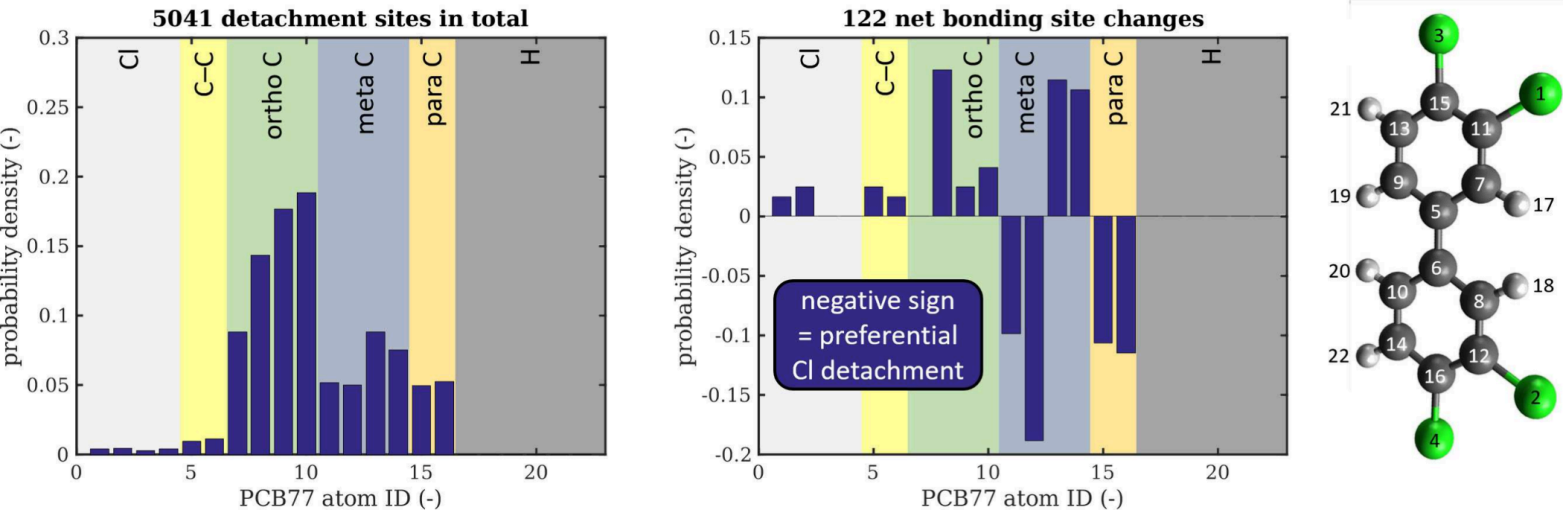
Probability density of Cl detachment (left) and net bonding site changes (center) under shear at 2100 K and 1.0 GPa as a function of the atomic identifier of an unaltered PCB 77 molecule (see legend on the right for which ID corresponds to which atom). A negative value in the differential plot (center) corresponds to preferential Cl detachment at that molecular site.

Finally, the chlorination and dechlorination events can be assigned to the carbon atoms making up the original biphenyl double ring. Since in the initial configuration, atomic indices within each molecule can be assigned to the ortho, meta, and para positions, one can produce probability density distributions, averaged over an entire shear simulation, for dechlorination, chlorination, and the difference between the two, see Figure 5. The result of this is a distribution that reveals from which to which positions on the aromatic ring the chlorine atoms preferentially move under shear at a given temperature and pressure. It has to be kept in mind, though, that this approach works only well as long as the aromatic structure remains intact and only the chlorine and hydrogen atoms shift. As soon as the aromatic rings are no longer intact, the results progressively lose their meaning.

In conclusion, it can be stated that we have developed a powerful and robust workflow that allows us to study the influence of temperature, confinement, pressure, and shear on the decomposition behavior of a given type of molecule in a deconvoluted way.

#### 2.2.3 Ab Initio Molecular Orbital Calculation

The advantage of MD is that in can treat larger system sizes and is inherently dynamic, thus we can observe the evolution of reaction processes over time. However, MD as an empirical model-based simulation uses empirical force fields (potentials), so it does not provide the same level of accuracy as quantum-based mechanical models that directly calculate the electronic structure of systems. Hence, to support the findings based on the outcomes of reactive MD, we also performed selected density functional theory (DFT) calculations. In order to aid the selection of a model compound for PCB 77, molecular descriptors were calculated for all chemicals. Additionally, the results from the reactive MD calculations suggested that several degradation pathways occur simultaneously. From reaction theory, the first step is to dissociate the carbon-chlorine bonds in the PCB 77 molecule. Among the four carbon-chlorine bonds in the molecule, two pairs are identical because of the molecular symmetry. Therefore, possible intermediates are carbon radicals at the C3- and C4-positions for which the bond dissociation enthalpy (BDE) values were calculated, among others.

The initial geometries of the molecules for the actual DFT calculations were preoptimized using the NWCHEM QM software.^27^ Several DFT functionals were explored (PBE0,^28^ BLYP,^29,30^ B3LYP^31,32^) in conjunction with different basis sets. After the performed set of test calculations, the hybrid PBE0^28^ functional and def2-TZVPPD^33^ basis set with the diffuse and polarization functions were chosen as the working model of theory. The PBE^34^ GGA functional with the dispersion correction was employed for the bulk and surface calculations under periodic boundary conditions using hard potentials in VASP.^35^ Convergence tests to determine the optimal value for energy cutoff and k-point grid were performed using High-Performance Computing (HPC) facilities. The convergence criteria on energy and gradient that should be fulfilled before convergence occurs for geometry optimization calculations were set to 10^–6^ and 10^–4^ a.u., respectively. The convergence thresholds for the single point energy minimization calculations were set to 10^–8^ a.u. Vibrational analysis was performed on all equilibrium structures, and all-positive Hessian eigenvalues were obtained for each optimized structure.

#### 2.2.4 Considerations on the Decomposition Temperature

Treatment of waste PCB by incineration is usually carried out at high temperatures ≈1000 K to prevent the formation of unintentional persistent organic pollutants such as dibenzodioxins.^36^ Our reactive MD calculations suggest that the reaction requires much higher temperatures (>1800 K) to initiate. It is frequently stated in computational chemistry that harsh conditions are chosen for minimizing the calculation time. Although the set of harsh conditions may seem practical, it requires some theoretical insight whether this is reasonable or not:

1. It is well established in chemical kinetics that the rate of a chemical reaction depends on the activation energy. As the activation energy increases, the reaction slows down. If a reaction occurs in a sequential multistep way, the unit reaction with the highest activation energy determines the overall kinetics. This step is defined as the “rate-determining step”. As reactive MD does not calculate the activation energy, an acceleration of the process using harsh conditions is not exact, but it may be convenient.
2. It is empirically well-known that a reaction whose activation energy is smaller than 80 kJ mol^–1^ can occur at room temperature.^37^ Since the available thermal energy in the system is ≈2.5 kJ mol^–1^ at room temperature (≈ 300 K),^38^ it seems insufficient for the reaction to take place. Here the Boltzmann equation estimates the ratio of molecules above 80 kJ mol^–1^ at room temperature is . This number is very small, but in the real world numbers of molecules on the order of Avogadro’s constant  are present, where substantial numbers of molecules are at the state of sufficient energy to exceed the activation barrier. On the other hand, computational studies are usually conducted with some 10^2^ molecules. Therefore, the number of molecules in the system with enough energy would be almost negligible.
3. In reactive MD calculations, all molecules are in the ground state, therefore different from the real world where Boltzmann theory applies. It is assumed, that to observe representative reactions, most of the present molecules has to be sufficiently activated leading to higher input energy requirements resulting into application of excessive conditions, such as temperature, pressure, and shear.

## 3 Results and Discussion

### 3.1 Thermal Studies in the Bulk System

In the first approach, we studied thermal degradation of the model compounds using a bulk simulation setup, i.e., periodic boundary conditions in all three spatial dimensions, through which atoms and molecules can move (no physical hindrance by simulation box walls). These thermal degradation studies resemble pyrolysis under inert atmosphere-like conditions (only molecules of interest are present), which is applied in the case of PCB elimination via incineration carried out at ≈1500 K.^39^

In the preliminary studies, molecules were subjected to constant temperatures of 300, 600, 900, 1200, and 1500 K to find out when the decomposition starts to occur. These simulations were carried out with 50 molecules of each studied chemical in an NVT ensemble, where the number of atoms, the volume, and the temperature are fixed, while the system pressure adjusts to the mentioned constraints. Note that the number of molecules being the same for each species here does not lead to any inconsistencies with the main results presented in this article (where we adjusted the number of molecules per system to keep the number of atoms approximately equal). These prescreening studies with brief simulation durations of 80 ps revealed that all studied chemicals appeared to remain highly stable and only exhibited minor dissociation corresponding mainly to a release of Cl due to C–Cl bond breaking. We concluded that the selected conditions, especially the short simulation duration and constant temperature were not sufficient to observe extensive decomposition. This precludes reliable qualitative and quantitative data analysis serving for degradation mechanism elucidation and does not enable the selection of a representative model compound of PCB 77 with similar stability performance for experimental validation.

Thus, to identify when the decomposition takes place to a sufficient extent, thermal degradation studies were carried out under continuously increasing temperatures up to 3000 K. This approach was optimized in several iteration steps until a reliable simulation setup could be established. The BO cutoff was optimized to a value of 0.6, which enabled us to capture all bond types of interest between the C, H, and Cl atoms present in the system, while avoiding registration of simulation artifacts, as discussed in the simulation setup section and the Supporting Information. The presented results are for thermal degradation studies with the temperature ramped linearly from 1–3000 K at a heating rate of 1.5 K/ps and for a duration of 2000 ps for all three chemicals of interest.

**Figure 6:**
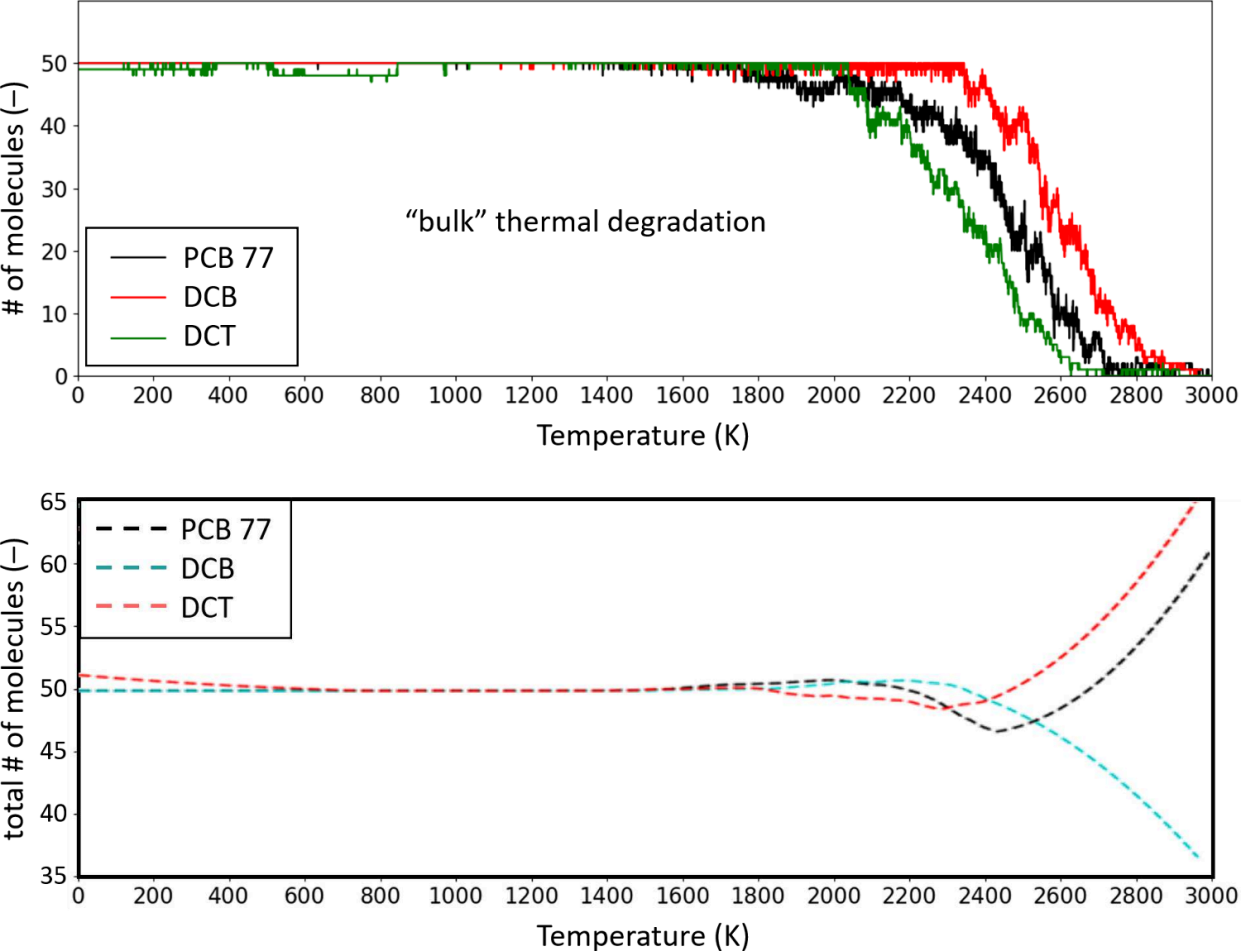
Top: Bulk thermal degradation of PCB 77, DCB, and DCT. The temperature was ramped linearly from 1 to 3000 K at a rate of 1.5 K/ps. Bottom: Total number of molecules (reactants and products) during the bulk thermal degradation of 50 molecules of PCB 77, DCB, and DCT. The temperature was ramped linearly from 1 to 3000 K at a rate of 1.5 K/ps.

As shown in Figure 6, top panel, the degradation temperature of DCT lies closer to the degradation temperature of PCB 77, hence these two chemicals have comparable stability under otherwise identical conditions. This constitutes one important consideration for its use as model compound for PCB in experimental studies. As seen, significant degradation of the studied chemicals occurs above 1500 K, which explains the lack of degradation species in the constant temperature simulation runs performed up to 1500 K. By contrast, under the temperature ramp conditions, all three chemicals are entirely degraded at the end of the respective simulation run.

Hence, the evolution of the total number of molecules in the system, i.e., the initial compounds and their degradation products, could be evaluated over the simulation run duration and plotted as a function of increasing temperature. At first, PCB 77 decomposition leads to the formation of larger degradation products as the number of molecules in the system drops, but this trend is soon reversed, and smaller degradation products than the initial molecule are formed toward the end of the simulation run. A similar trend can also be seen for DCT, but the evolution of the number of molecules during the pyrolysis of DCB shows an opposing trend, where after a minor initial increase in the number of molecules, reflected by formation of smaller degradation products, larger degradation products are continuously produced until the simulation ends. These findings are presented in Figure 6, bottom panel.

**Figure 7:**
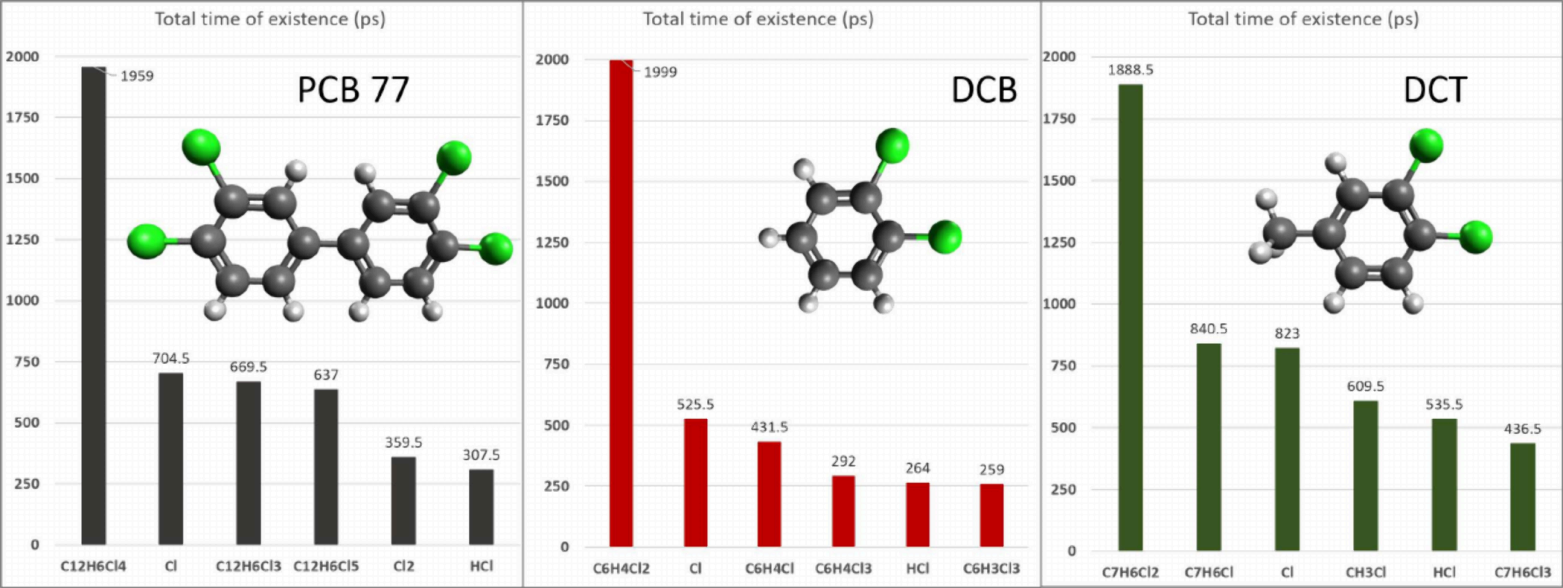
Total time of existence of the dominant molecular species during bulk thermal degradation of PCB 77, DCB, and DCT.

During each of these simulation runs, hundreds of species were registered for each studied chemical, as the molecules were decomposed entirely at the end of the temperature ramp. It has to be kept in mind that the produced chemical species are often short-lived and that they might occur just once throughout the entire simulation run. Thus, in order to identify the main degradation products, an evaluation based on the total time of existence over the 2000 ps simulation run was carried out. This revealed that the Cl radical is mostly present in the system, together with the corresponding dechlorinated molecule of the initial chemical, followed by the initial molecule with one more bonded Cl atom as well as HCl. In the PCB 77 simulation run, Cl_2_ was was also among the most common species, probably due to a higher abundance of C–Cl bonds compared to the other studied chemicals. Only in the case of DCT, also CH_3_Cl was formed, indicating the release of the methyl group. This analysis shows the preferential formation of particular species, but does not take into account their abundance. The presented distribution of the detected species is shown in Figure 7.

**Figure 8:**
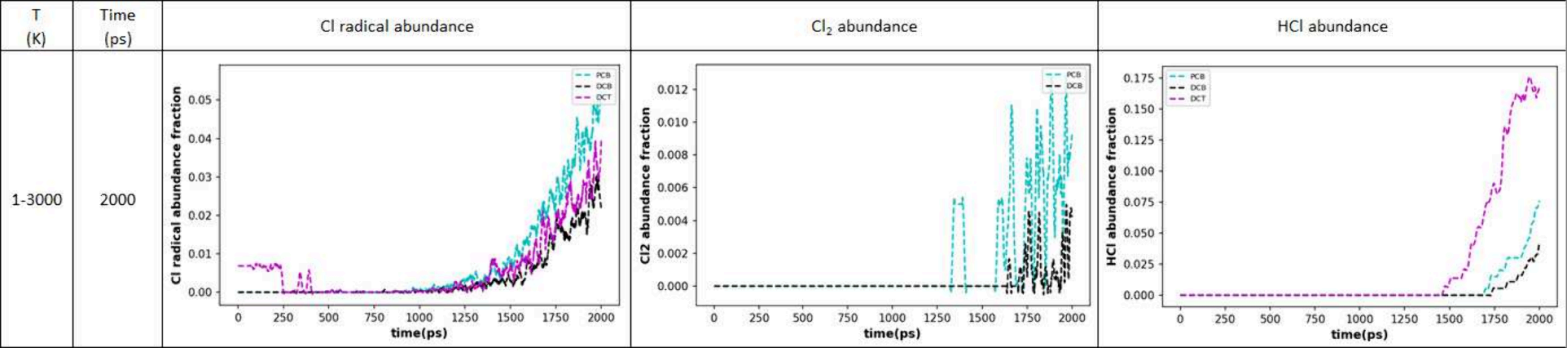
Fractional amounts of the Cl radical, Cl_2_, and HCl for the studied PCB 77, DCT, and DCB chemicals for the temperature range 1–3000 K under 1.5 K/ps heating rate and 2000 ps duration. Chemicals are represented by the following colors: PCB (blue), DCB (black), DCT (purple). The odd behavior of the Cl radical abundance for DCT at *t* < 250 ps corresponds to repeated dithering of only two molecules between the original C_7_H_6_Cl_2_ and C_7_H_6_Cl + Cl^•^ at low temperatures.

The above qualitative observation corresponded well with the aim to decompose aryl-chlorides, focusing on the dissociation of the inherently stable aromatic C–Cl bond, while the released Cl should be preferentially stabilized into stable inorganic (and therefore less toxic) compounds. Hence, the C–Cl bond dissociation reflects the degradation extent of the initial molecules. For this aim, the occurrence of Cl radicals was evaluated. Additionally, the formation of inorganic compounds (those without C atoms) was plotted as the abundance of the formed Cl_2_ and HCl products. To compare the yield of these species, their abundance was normalized by the total number of Cl atoms available in each studied system. As 50 molecules were used in each simulation, the total number of Cl atoms was 200 for PCB 77 and 100 for DCT and DCB. The evolution of the Cl radicals, Cl_2_, and HCl in this quantitative manner is shown in Figure 8.

As can be seen, slightly more Cl radicals are formed in the case of PCB 77, which can be explained by the fact that this molecule has twice as many Cl atoms compared to DCT and DCB. In the case of DCT, the amount of Cl radicals is slightly higher than for DCB and closer to the PCB 77 values. In all cases, the fraction of Cl radicals increases sharply with time and hence with temperature, thus temperature is an important factor in C–Cl bond breakage. However, purely thermal degradation of PCBs, especially in the presence of an oxygen source as in an industrial incineration process, can lead to the formation of even more toxic products such as dioxins.^40^ Thus, other means of C–Cl bond breakage should be explored to facilitate the safe decomposition of aryl-chlorides, such as pressure and shear effects, the influence of which is further presented in the following section. The formation of Cl_2_ is negligible compared to the abundance of HCl. DCT and DCB differ in the formation of these inorganic products, as Cl_2_ was not detected in the DCT system at all. However, its abundance was negligible and highly fluctuating compared to HCl formation. Especially in the case of DCT, it produces significant amounts of HCl of increasing trend (stabilized product formation), which is considered beneficial for the capture of Cl atoms into stable inorganic products. However, in the case of PCB 77 and especially DCB, the amount of HCl is less significant, so other means of Cl capture might be explored.

Based on these thermal studies and the comparison of the two model compounds to PCB 77, the stability under identical conditions, the trend of decomposition toward buildup of smaller degradation products, and the abundance of Cl radicals and HCl is better reflected by DCT, making it a more suitable candidate for experimental studies. It is clear that the applied temperatures and heating rates in the simulations are exaggerated compared to experimental or industrial conditions. As mentioned earlier, the necessity for this can be attributed to the high activation energies required for the reactions to be initiated and that most of the present molecules in the system need to be activated in order to obtain the main degradation products and pathways in a representative way. Without this excessive energy provided to the system, the approach would not be able to capture the degradation reactions within a reasonable simulation time. It should be re-emphasized that we do not attempt to evaluate the exact degradation temperatures of the studied compounds, but rather focus on finding the similarities between the degradation behaviors of the studied chemicals in order to select the most suitable candidate to replace PCB 77 in experimental studies.

### 3.2 Heating, Pressurizing, and Shearing in a Confined Gap

To explore the contributions of additional conditions to aryl-chloride decomposition, such as pressure and shear, the simulation cell was altered so that molecular movement through the simulation box boundary in *z*-direction is prevented, thus creating a gap-like condition. The aim was to keep the gap size the same for all studied chemicals, to study the decomposition of the chosen chemicals under similar geometrical conditions, in particular the shear strain rate, and thus enabling direct comparisons. As the model chemicals differ in size, we kept the total number of atoms in the system as close to 1100 atoms as possible. This leads to a different number of molecules in the system used for each chemical: 50 for PCB 77, 73 for DCT, and 92 for DCB. Note that the gap thickness still slightly varies as a function of the applied pressure especially for DCT, but it is comparable between the compounds, as can be seen in the last 2 rows in Figure 2.

A considerable set of parameters was used to determine the susceptibility of PCB 77 toward degradation and to compare it to the other model compounds. As learned from the thermal studies in the bulk system, under constant temperatures up to 1500 K, the studied chemicals remain stable except for occasional instances where the C–Cl bond breaks and reforms repeatedly. Hence, the temperatures of 1800 and 2100 K were applied, the studied pressure was selected at 0.5, 1.0, and 2 GPa while the shear strain rate remained constant at 10^10^ s^–1^. Viewing the number of initial molecules throughout the simulation run, we concluded that the initial molecules already degraded considerably during preconditioning (setting of temperature followed by pressure) except for the mildest combination of applied conditions at 1800 K, 0.5 GPa, and 10^10^ s^–1^ shear strain rate, as shown in Figure S3 in the Supporting Information, where the red dotted line illustrates the number of initial molecules in the system for better visualization of the extent of degradation.

This observation can be explained by the chosen approach for careful preheating of the system aiming to keep the molecules intact, which however resulted in a long duration of the preconditioning step lasting over 2 ns, while the pressurizing phase lasted up to 0.5 ns. This long preconditioning in comparison to shearing (simulation run) phase of 1.2 ns was enough to induce decomposition of the studied molecules and so hindered the evaluation of their degradation from its initial point. This limits the capability to observe all occurring chemical species evolved throughout the degradation process. At a temperature of 1800 K, the decomposition once initiated, remained at a rather constant level as is reflected by the almost stable amount of initial molecules throughout the simulation runs (horizontal trend line), apart from 0.5 GPa where most of the degradation took place during the shearing phase of the simulation. However, for the higher pressures of 1 and 2 GPa, most of the degradation occurred already during the preconditioning phase and did not significantly proceed any further, which reflects the contribution of the increased pressure on the degradation extent at a constant 1800 K. Between the 0.5 and 2 GPa, there is a ≈40% increase in degradation for both PCB 77 and DCT, but only 25% for DCB. The percentage of lost initial molecules is similar between PCB 77 and DCT, while DCB remains the most stable among the studied chemicals.

Although the decomposition of the studied chemicals is already initiated in the preconditioning phase at 2100 K, the decomposition rapidly proceeds further throughout the shearing phase, as indicated by strong drop in the amount of initial molecules. This might suggest a contribution of the shear strain rate on the decomposition, as in the case of 1800 K and 0.5 GPa, or may simply be the result of decomposition proceeding further throughout the simulation time due to the extensive provided energy. It was again observed that the degradation extent between PCB 77 and DCT is very close compared to DCB. At 2100 K, the contribution of the increase in pressure from 0.5 to 2 GPa was lower compared to the 1800 K simulation runs and ranged from ≈10–30%.

As there was indication of a shear strain rate contribution to the decomposition, it was further studied by comparison with simulation runs without applied shear, as exemplarily presented in Figure S4 in the Supporting Information for the PCB 77 case at 1800 K. From the observed amount of PCB 77 molecules throughout the simulation runs, it can be seen that shear does not significantly contribute to the degradation extent, and the trend lines of degradation remain similar and practically constant.

**Figure 9:**
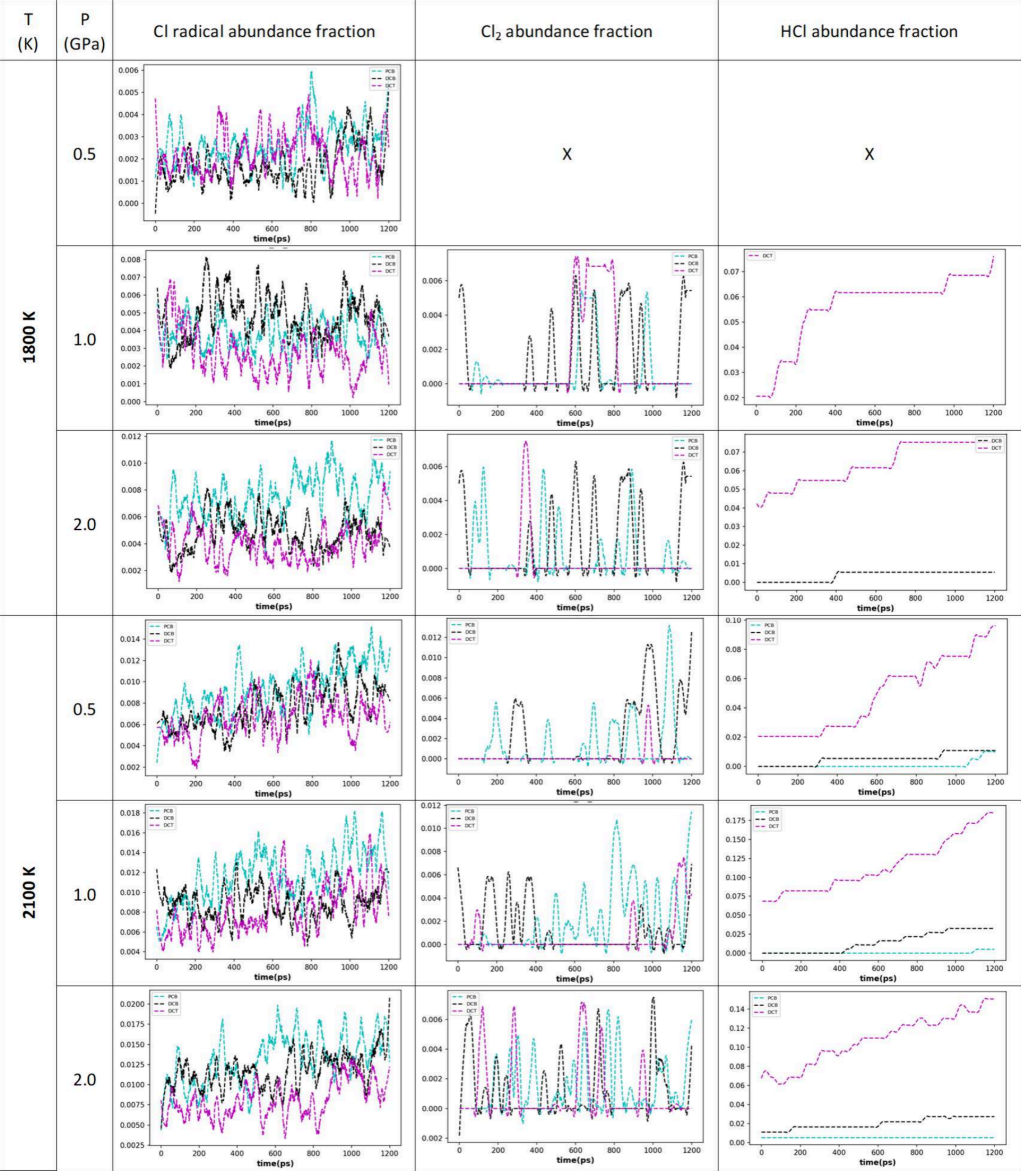
Abundance fractions for Cl radicals, Cl_2_, and HCl during thermally and pressure-driven decomposition of PCB 77 (cyan), DCB (black), and DCT (magenta).

When considering the fractional abundances (normalized per available Cl atoms in the selected number of molecules) of released Cl from C–Cl bond breakage as well as of Cl_2_ and HCl as desired inorganic products, the same trends were observed as in the bulk thermal studies. The Cl abundance fluctuates throughout the simulation runs, but shows an increasing trend under harsher conditions, leading to increasing and repeatable C–Cl bond breaking and formation. The abundance of Cl_2_ compared to HCl is minor, and almost absent for DCT. Its fluctuation indicates that Cl is not preferentially stabilized in this form. On the other hand, HCl abundance is significant for DCT and shows an increasing trend throughout the simulation run which indicates the stabilization of Cl into this compound, but for of PCB 77 and DCB, the abundance is minor. The results are presented in Figure 9. Further evaluation of additional decomposition products concluded that tens of products are formed during the 1800 K runs, while hundreds of products are formed during 2100 K simulations. When looking at chemistry overlaps between these degradation products (based on SUM formula comparisons), both DCT and DCB reflect the chemistries of PCB 77 degradation products on the same level, with the exception of the most severe conditions at 2100 K and 1.0 and 2.0 GPa, where DCB results in a better match. However, due to substantial degradation already having taken place in the preconditioning phase, further elucidations were omitted as the initial degradation phase was not considered in the simulation output data.

As can be concluded, temperature contributes the most to the degradation of the studied aryl-chlorides, while the applied shear has only minor influence, likely due to the rather planar structure of the studied molecules. To further investigate the contribution of the increased pressure on the amount of degradation, pressures of 3, 4, and 5 GPa were added to the tested temperatures of 1800 and 2100 K, while also the temperature at 1500 K was selected to observe if the pressure increase can initiate degradation already at this temperature level. All simulation runs were carried out at an applied shear strain rate of 10^10^ s^–1^ for further consistency and comparison of the obtained results with the previous ones. As DCT had so far been found to be most similar to PCB 77 based on its degradation under the same applied conditions, DCB was excluded from these higher pressure studies. Furthermore, to avoid the considerable degradation already taking place in the long preconditioning phase, we applied an alternate modeling approach of “shock-heating” combined with pressurization. The preconditioning step of thermalization and pressurization was thus shortened by about 120 times, while the length of the simulation run corresponding to the shearing phase was kept the same at 1.2 ns. This enabled the preservation of the initial number of molecules in the system until the start of shearing (data not shown) even under the most severe conditions (highest temperatures and pressures). Therefore, if the correct gap height is known for a given combination of molecule type and number as well as temperature to maintain a selected pressure, this combined shock-heating and pressurization approach is preferential to a careful preheating phase of long duration due to better chance of degradation pathway assessment, as the system is preserved intact prior to the data acquisition during the shearing stage.

As also observed previously, the stability (evaluated as the percentage of initial molecules remaining in the system) of PCB 77 and DCT is similar under the same applied conditions, as shown in Figure S5 in the Supporting Information. Hence, DCT is a promising model compound choice for experimental studies aiming to validate computational findings even for toxic and difficult-to-handle materials such as PCBs. It can be concluded that a pressure increase itself does not have the same capability to drive the decomposition to a significant extent as compared to a thermal energy increase at the same pressure level. Additionally, the increase of pressure up to 5 GPa at 1500 K was not sufficient to cause degradation of either DCT or PCB 77. A significant increase in thermal energy (≈300 K) is required to enable degradation to take place. The largest contribution of pressure to degradation was observed for the pressure increase from 1 to 2 GPa at 1800 K for PCB 77. The simulation run for the DCT at 1500 K and 3 GPa failed and was not included, but we expect that no degradation took place, as this was also the not case under higher pressures of 4 and 5 GPa at this temperature.

**Figure 10:**
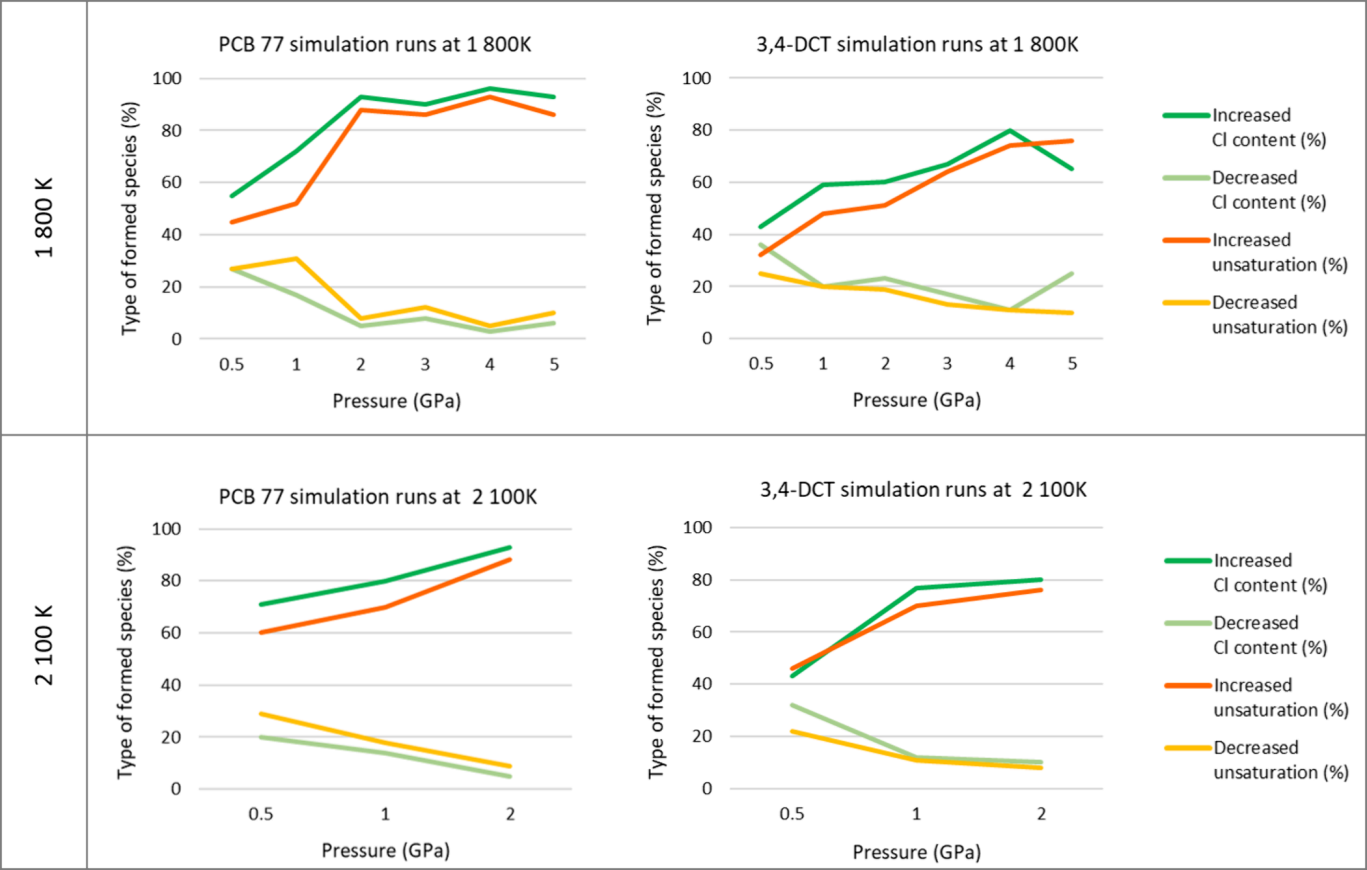
Overview of *T* and *p* dependence of Cl content and unsaturation for PCB 77 and DCT.

For a global evaluation of the degradation trend where hundreds of chemical species are formed within one simulation run, we carried out an assessment of the type of formed chemical species. It was of interest to assess if the chlorination extent decreases and if the saturation of molecules changes, indicating a decrease of aromaticity (pi bonds in resonance resulting in increased stability compared to saturated compounds with single bonds), while both trends would be favorable from the toxicological point of view. For the approach to assess the aromaticity (degree of unsaturation) of the molecules, the ring double bond equivalent (RDBE) calculation was applied^41^ to the generated SUM formulas of the formed chemical species. As the name states, it determines the number of rings and double bonds in the molecule by utilizing the molecule’s chemical formula stating the type and number of elements. Such calculations are also commonly applied in high-resolution mass spectrometry to aid in molecular structure determination. The analyses of these changes are presented in Figure 10. It was evaluated that degradation products containing more Cl-bonds than in the initial molecule are formed. This can be explained by the fact that once the C–Cl bond is broken, the formed Cl can react with other molecules in the system and it less frequently leads to the formation of inorganic chlorides such as Cl_2_ and HCl, which would be beneficial due to their significantly lower toxicological impact compared to persistent toxic PCBs. Additionally, also the unsaturation increases with the severity of the applied conditions, i.e., more double bond or ring systems are formed. Furthermore, based on the RDBE calculation, about 40–50% of all species formed throughout the simulation run are radicals, which affects the calculation of the RDBE value reflecting the degree of unsaturation.

**Figure 11:**
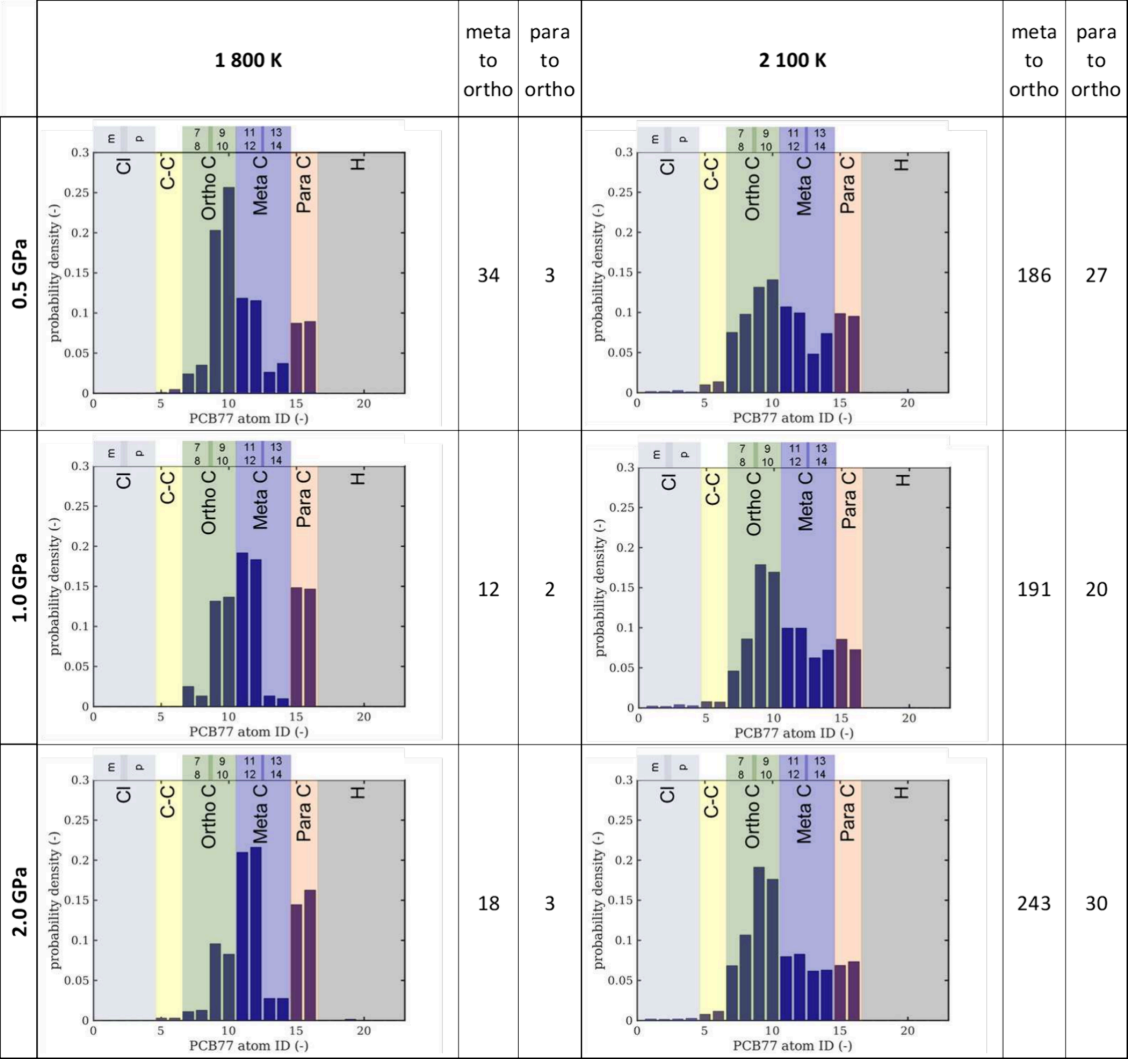
Probability densities for possible Cl bonding sites (ortho, meta, para) for PCB 77 as a function of *T* and *p*. Total numbers of transitions from meta to ortho and para to ortho positions are given for each set of parameters.

We also quantified how many times and when each individual Cl atom changed its bonding partner, also capturing nonbonded Cl atoms, as well as identifying particularly reactive Cl atoms using the explicit atom labeling approach introduced in Figure 4. The results are presented in the Cl reactivity maps for each of the 200 Cl atoms which are labeled in 50 molecules of PCB 77 within one simulation system, as shown in Figure S6. The focus was laid on the temperatures of 1800 and 2100 K combined with pressures at 0.5, 1, and 2 GPa, as higher pressures did not lead to any significant increase of degradation. Even when hundreds to thousands of reactions (Cl bonding partner changes) occur, the variety of chemical species formed during these simulation runs remains with the order of tens, a 3-fold decreased compared to the previous slow preheating approach, also resulting in less degradation of PCB 77. The Cl reactivity maps and the low number of chemical types formed considering the large number of bonding partner changes leads to the conclusion of repeatable stages of bond breaking and formation resulting in the same chemical types. Although all of the PCB 77 molecules remain intact at the simulation end (based on the generated SUM formula) in the 1500 K simulation runs, the first decomposition already seems to take place, as some hundreds of Cl bonding partner changes occur (data not shown) due to repeated C–Cl bond breaking and formation. If many different radicals are formed simultaneously, this can still lead to the formation of different tetra-chlorinated PCB isomers that retain the same SUM formula as PCB 77. Hence, further data processing was carried out to obtain the information on the Cl positions on the biphenyl ring. To identify the Cl-position, the individual atoms in PCB 77 were assigned ID numbers so the Cl bonding partners could be visualized. They are bonded to carbon atoms in the meta and para positions only, as shown in Figure S7 in the Supporting Information. To compare the results among different applied conditions, the *y*-axis in the generated histograms is expressed as a probability density, normalized to obtain an integral value of 1 over the absolute values. We found that already at the next temperature of 1800 K at 0.5 GPa pressure with a shear strain rate of 10^10^ s^–1^ we could observe that the positions of Cl are redistributed among all available carbons on the aromatic ring as shown in Figure 11. Not only the carbons in the meta position originally bonded to Cl (IDs 11, 12), but also the carbons in the meta positions bonded to hydrogen (ID 13, 14) can become a Cl bonding partner. The distribution of the Cl bonded to these meta positions becomes more equal with a temperature increase to 2100 K. In addition, the carbon atoms in the ortho position become Cl bonding partners with a clear preference to the carbon atoms with ID 9, 10 (rather than to ID 7, 8), where the carbon in the neighboring meta position is not bonded to the Cl atom. With increasing pressure, reduced molecular mobility limits Cl transfer to these sites, but at 2100 K, higher thermal energy overcomes this restriction, resulting in a more uniform bonding distribution. At this higher temperature, the carbon atoms in the ortho position even become the predominant Cl bonding partner compared to the originally occupied meta and para positions in the intact PCB 77 molecule. We were further able to quantify how many Cl atoms originating from the meta and para positions, respectively, were contributing to the bonding of the carbon atoms at the ortho position. Clearly, the Cl atoms from the meta position were prevalent. Under more severe conditions, a small fraction of carbon atoms originally participating in the biphenyl bond bonded to a Cl atom, indicating a split of the biphenyl bond. A minor indication of Cl–Cl bonding leading to Cl_2_ formation can also be seen for the most severe conditions studied. The preferential sites of Cl atom dissociation and its following bonding sites provided further insights into the degradation of PCB 77. The bonding positions of Cl atoms are of high importance from the toxicological point of view, as the occupation of the ortho position forces the molecule into noncoplanar conformation, which leads to a significantly decreased toxicity.

The complex results from our reactive MD simulation runs where tens to hundreds of degradation products are formed within 1 ns duration required development of several postprocessing approaches as discussed above. Apart from observation of general trends, the focus was oriented onto the most abundant and longest persistent degradation products in order to outline the main degradation pathways under consideration of fundamental chemical reaction theories. It must be also kept in mind that most of the chemical species registered during the simulation time are short-lived or they appear in very small abundances. Based on the reactant/product heat maps introduced in Figure 3, we identified tri- and penta-chlorobiphenyl products as the most abundant degradation species of PCB 77 under all studied conditions, and an analogous degradation trend was revealed also for studied DCT and DCB model chemicals. Hence, higher chlorinated species are formed as the Cl radicals bond to the original initial molecules.

### 3.3 Density Functional Theory Calculations

Structure–property relationship calculations aimed to confirm the suitability of DCT as model compound for PCB 77 as concluded from our reactive MD studies. Hence, the resemblance between PCB 77 and its selected model compounds DCT and DCB was based on gas phase calculations of various electronic structure properties selected to be insensitive to the spatial orientation of the molecules. Selected molecular descriptors were evaluated based on the quantitative estimation of similarity using the mean absolute percentage deviation (MAPD). The MAPD expresses the accuracy as a ratio defined by the formula:
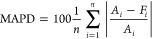
1where *A*_*i*_ is the PCB 77 calculated property value and *F*_*i*_ is the DCT or DCB forecast value of the same property. The results of comparative analysis with the MAPD values in percentages are presented in Table S1 in the Supporting Information. The bond lengths and bond dissociation energy (BDE) values are relatively of the same order of magnitude for the all considered compounds. The important properties such as ionization potential and HOMO–LUMO gap, chemical hardness, electrophilicity, electrophilic susceptibility, polarizability, and magnetizability favor the DCT compound. Also, the overall averaged MAPD yields a smaller error for the DCT compound, suggesting DCT as a promising model compound candidate to substitute PCB 77 in experimental studies.

The DFT method was applied to ascertain the observed preference of Cl dissociation positions based on calculated bond dissociation energy (BDE). The BDE for each carbon–chlorine bond in PCB 77 was calculated as 365.63 kJ mol^–1^ (for C3–Cl) and 367.73 kJ mol^–1^ (for C4–Cl), by *ab initio* molecular orbital calculation using Gaussian 16W software^42^ at unrestricted hybrid DFT method UB3LYP/6-31+g(d,p) level.^30,31^ The B3LYP-D functional is well-suited for treatment of benzene.

### 3.4 Discussion on Reaction Pathway and Mechanism

The following statements about the degradation of PCB 77 can be formulated based on the obtained qualitative and quantitative results from our MD simulations. The effect of three studied influence factors on PCB 77 decomposition follows the order: temperature > pressure ≫ shear. Considering the individual studied cases, the shear contribution is almost negligible.

**Figure 12:**
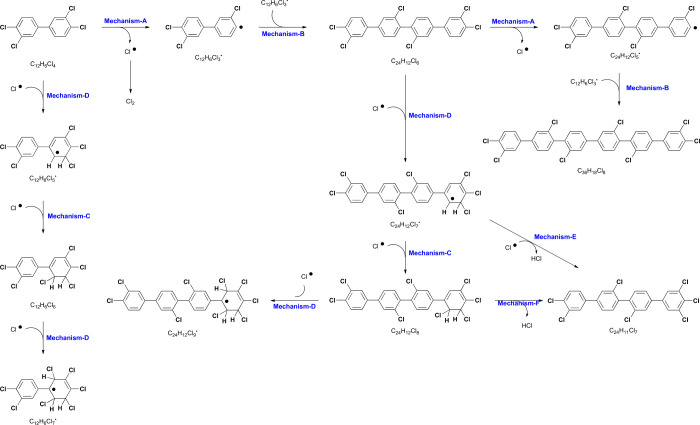
Major degradation pathways of PCB 77 from reactive MD simulations, irrespective of isomers.

To understand the complicated reaction pathways, we first focused on the abundant products in the system. Then, we considered stepwise reactions as the combination of the unit reaction according to the theories of organic reactions. To eliminate the complexity, Figure 12 depicts structures irrespective of the isomers (the reaction point on the biphenyl ring). A clarification of the regioselectivity of the reaction would require an evaluation of the energy barrier for each step. According to Boltzmann theory, the probability distribution (*q*) of particles in a system depends on the state’s energy (*E*) and temperature (*T*), *q* = *e*^–Δ*E*/*kT*^, where *k* is the Boltzmann constant. While there are considerable differences in the isomeric ratio for the radical intermediates (≈1:0.4), those for the corresponding hydrogen adducts are nearly equal. As reactive MD does not calculate the transition state, the reaction mechanism will be discussed irrespective of possible isomers hereafter.

**Figure 13:**
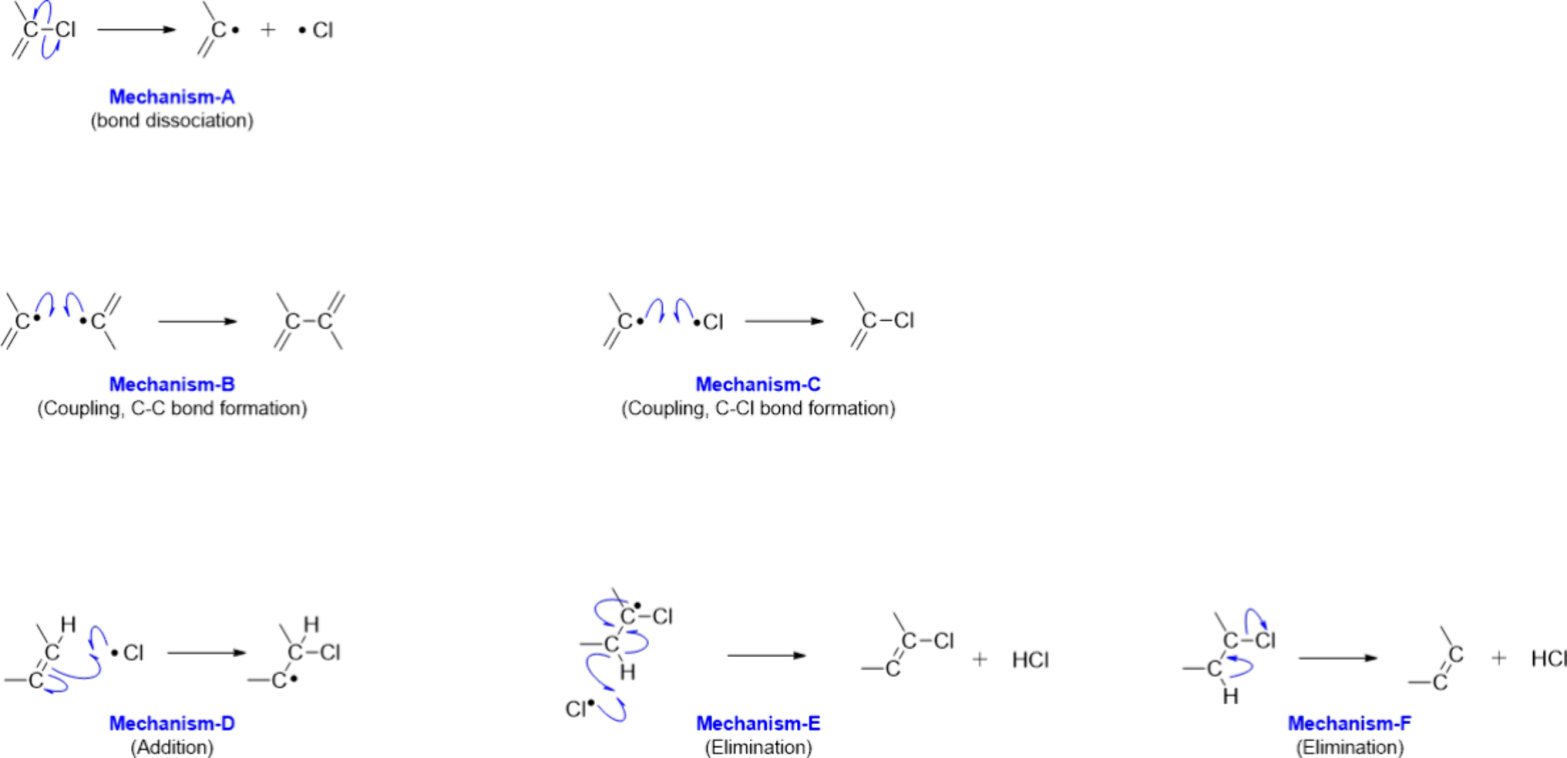
Mechanism of unit reaction in degradation pathways of PCB 77.

Apart from the formation of inorganic chlorides (such as HCl and Cl_2_), to our surprise, the addition of chlorine (biphenyl with more than 5 chlorine atoms) and the oligomerization of the ring system (more than 4 benzene rings) were suggested by the results. These products are well explained by the radical reaction mechanism, as illustrated in Figure 12. The reaction begins with the dissociation of one of the C–Cl bonds (Mechanism A in Figure 13), yielding a trichlorobiphenyl radical (C_12_H_6_Cl_3_^•^) and a chlororadical (Cl^•^). The coupling of the radical through Mechanism B results in a tetraphenyl species (C_24_H_12_Cl_6_), which further react through stepwise Mechanisms A and B to form multiple-ring moieties, such as C_36_H_18_Cl_8_.

The addition of the chlororadical (Cl^•^) to the double bond in benzene (Mechanism D) provides an additional chlorine atom on the biphenyl ring. At the same time, a carbon radical is formed on the other carbon of the double bond. The product, a pentachlorobiphenyl radical (C_12_H_6_Cl_5_^•^), can couple with a chlororadical through Mechanism C, yielding a hexachlorinated moiety, which is further chlorinated through Mechanism D. Similarly, chlorination occurs on the tetraphenyl moiety (C_24_H_12_Cl_6_) through Mechanisms D and C. The elimination reaction (Mechanism E) of a hydrogen atom in C_24_H_12_Cl_7_^•^ by a chlororadical yields heptachlorotetraphenyl (C_24_H_11_Cl_7_), which could lead to an octachlorotetraphenyl moiety (C_24_H_10_Cl_8_) through the addition of HCl (Mechanism D). Addition of a chlororadical is also possible for multiring moieties.

Summarizing the above, the degradation of PCB 77 under varying conditions elucidates a cascade of reactions dominated by radical-driven pathways that facilitate both chlorination and oligomerization processes. The results reveal how specific mechanistic steps, including successive C–Cl bond dissociation and radical coupling, contribute to the formation of multiring and highly chlorinated species. This highlights the intricate interplay of factors such as temperature, pressure, and chlorination mechanisms, underscoring the complex pathways PCB 77 may undergo in reactive environments. Further investigation into regioselectivity and transition states could enhance our understanding of these degradation mechanisms and inform approaches for effective PCB remediation.

## 4 Conclusions

In this study, reactive molecular dynamics was used to investigate the thermal reactions of polychlorinated biphenyls (PCBs) and their potential to produce multiring moieties and more chlorinated biphenyls, which may increase the risk of toxicity. The analysis of reaction pathways and mechanisms highlighted the importance of scavenging the chlororadical or deactivating the reactive species formed during the process. Effective Cl scavengers such as metals will be considered in future work to mitigate this risk.

The accuracy of simulation methods in predicting chemistries was also discussed. While exact chemistries may not always match, reproducing trends toward less harmful degradation products of the same chemistry class can decrease the demand for experimental trials. Analytical results will be used to validate the accuracy of the predicted chemistries.

One limitation of the study is that the applied ReaxFF potential cannot currently be used to involve metallic surfaces as a further important factor influencing the degradation of the studied chemicals. Prior to this, ReaxFF must be parametrized using DFT calculations to ensure reliable metal/organic molecule interactions. As a consequence, the released Cl radicals cannot be stabilized into organo-metallic or metallic salts, as assumed. Additionally, the study found that pressure can significantly induce degradation, while the effect of shear is minor, most probably due to the planar geometry of the studied molecules.

In summary, the study suggests that thermal reactions of PCBs can be complicated and produce potentially harmful degradation products. Effective scavenging methods, such as the use of metals, will be considered in future work to mitigate this risk. Furthermore, while simulation methods may not always accurately predict exact chemistries, they can be used to reproduce trends toward less harmful degradation products of the same chemistry class, reducing the demand for experimental trials. Finally, the study highlights the importance of considering pressure and shear effects on the degradation of PCBs.

## Data Availability

Any data beyond that already included in the Supporting Information will be made available by the corresponding author upon reasonable request.

## Supporting Information Available

Additional information on “Sanity checks and system preparation”, including bond order histograms and pressurization curves to obtain the correct gap thickness. Time development of lost molecules (PCB 77, DCT, and DCB) due to thermal loading. Influence of the shear rate on the decomposition of PCB 77. Bar chart on the similarity of the decomposition of PCB 77 and DCT. Reactivity maps for Cl atoms in PCB 77 systems. Probability density for Cl being bonded to the meta and para positions. Table with mean absolute percentage deviation (%) of DCT and DCB relative to PCB 77 property values (PDF)
