# Supplementary material for: Breaking Down Polychlorinated Biphenyls and Aryl Chlorides: A Computational Study of Thermal-, Pressure-, and Shear-Induced Decomposition
Source: J Phys Chem A. 2025 Feb 27;129(10):2568–83. doi: 10.1021/acs.jpca.4c08086 (PMC11912490; doi:10.1021/acs.jpca.4c08086)
Supplement: Supplementary file 1 — jp4c08086_si_001.pdf [file jp4c08086_si_001.pdf]

Supplementary Material for Breaking Down Polychlorinated Biphenyls and Aryl Chlorides: A Computational Study of Thermal, Pressure-, and Shear-Induced Decomposition

L. Pisarova[a], O. A. Loboda[a], I. Minami[b], S. J. Eder[a,c,*]

[a]*AC2T research GmbH, Viktor-Kaplan-Straße 2/C, 2700 Wiener Neustadt, Austria*
[b]*Department of Engineering Sciences and Mathematics, Division of Machine Elements, Luleå University of Technology, SE-97187 Luleå, Sweden*
[c]*Institute for Engineering Design and Product Development, TU Wien, Lehárgasse 6 – Objekt 7, 1060 Vienna, Austria*

Sanity checks and system preparation

In the first stage, several sanity checks (stability of molecules, proper molecular geometries, density, total energy conservation, etc.) were performed on the "bulk" system, i.e., a system periodically replicated in all three spatial dimensions, under ambient conditions using the NPT ensemble (300 K, 1 atm). This was done by monitoring the energies and the forces in the system, as well as by evaluating the ReaxFF bonds tables, which gave out any atom-atom interaction with a bond order (BO) greater than 0.3 as an existing bond every 1 ps. From these data, tables of the developing molecular species along a simulation timeline can be produced, based on a possibly better adapted BO threshold to eliminate any simulation artifacts. The BO threshold value is crucial for a reasonable determination of the degradation extent, i.e., to differentiate between bonded and non-bonded species. Thus, great care was taken to determine the proper threshold by observing the BO histograms of the bonds present in the system. Additionally, bonds that might be formed during aryl-chloride decomposition, namely Cl–Cl, H–Cl, and H–H, were considered. As all of these bonds lay above the BO value of 0.6, this value was used as BO threshold in all following evaluations, see Fig. S1 for the corresponding histograms.

After it had been established that the bulk system is properly set-up, that the molecules remain stable under mild conditions, and that the dynamics conform with the canonical NVT ensemble, the purely thermal degradation studies were performed on the three studied chemicals. Temperature ramps from 1–3000 K were applied at a heating rate of 750 K/ns for the duration of 2 ns. After determining that no relevant chemical changes occur below 1500 K within the applied short time spans observable by reactive MD simulations (further discussion in section 3.4 of the main text), we selected the system snapshots at temperatures of 1500 K, 1800 K and 2100 K to serve as the basis for compression simulations.

Hence, the two periodic system boundaries normal to the z direction were replaced with flat repulsive (Lennard-Jones 9–6) walls to mimic confinement in an infinite two-dimensional gap of constant thickness. In the compression runs, the z dimension of the respective system was linearly reduced from 2.8 nm to 1.4 nm over 0.8 ns, which corresponds to a compression speed of 1.75 m/s. While the increasing system pressure was recorded and plotted over time, it has to be kept in mind that as soon as the system is sheared afterwards along the x direction, the molecules have opportunity to rearrange laterally, which will lead to a decrease in the system pressure. We therefore conducted brief shear simulations on system snapshots, equidistantly spaced throughout the respective compression simulation, to allow the system pressure to relax to the value

*Corresponding author
Email address: stefan.j.eder@tuwien.ac.at (S. J. Eder)

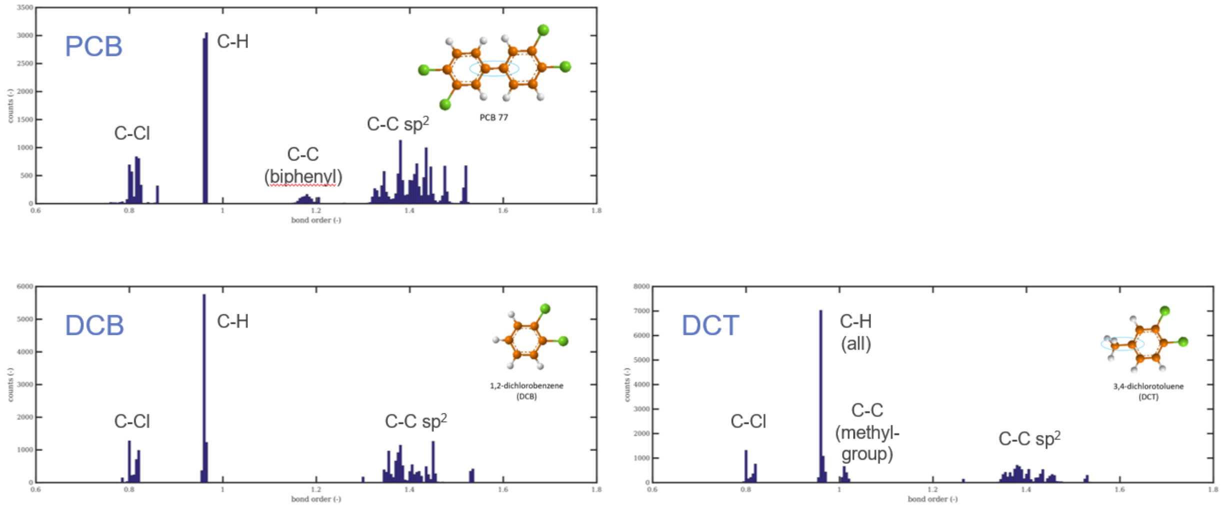

Figure S1: Bond order (BO) histograms for all considered initial molecular species PCB-77, DCB, and DCT, to justify the selected lower BO threshold of 0.6.

it would assume under shear for a particular gap thickness. Figure S2 illustrates this approach for the three selected molecules at the representative temperature of 1500 K. The shear-equilibrated and time-averaged pressures are shown as red circles, which are connected by a blue spline interpolant. Intersecting the interpolant with the desired system pressures for the shear simulations, intended for data production and evaluation, yields the correct time step from the compression simulation that will serve as the initial configuration for the shear runs (marked along the interpolant as black boxes). The workflow illustrated in Fig. S2 led to a range of system heights as a function of chemical structure (molecular size), temperature, and desired system pressure.

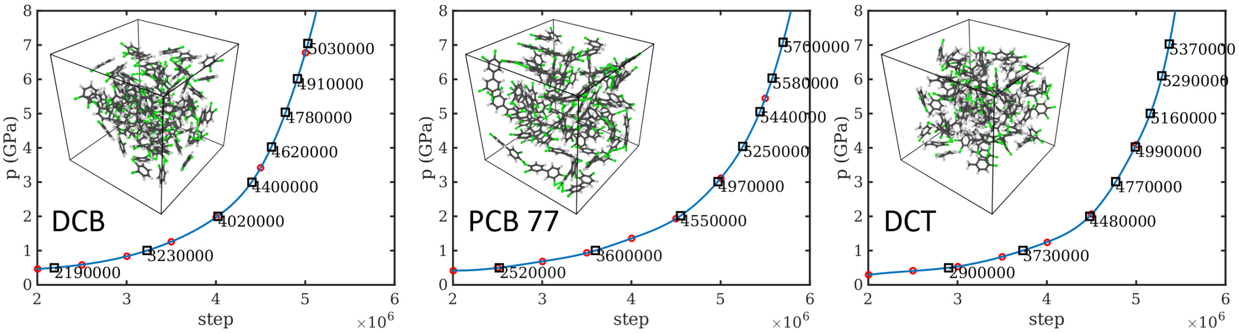

Figure S2: On how to estimate the correct gap thickness representing a desired normal pressure. Short shear simulations were carried out at a regular gap thickness interval, allowing the pressure in the system to equilibrate. The equilibrium values are represented as red circles. The blue curves are cubic spline interpolants, yielding the time steps of the compression simulation that best represent the desired pressures of 0.5, 1, 2, 3 GPa, etc. (black boxes annotated with time step number)

	T (K)	0.5 GPa	molecules lost (%)	1.0 GPa	molecules lost (%)	2.0 GPa	molecules lost (%)
**PCB 77**	1800	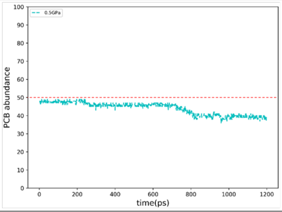	24	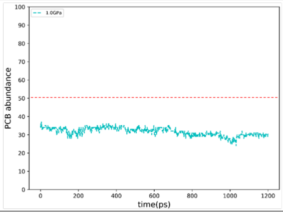	38	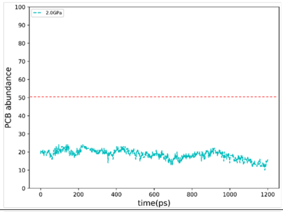	62
	2100	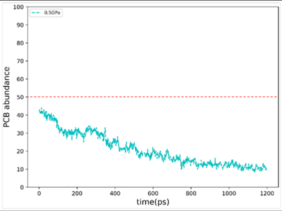	80	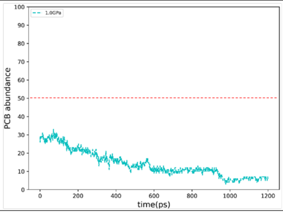	88	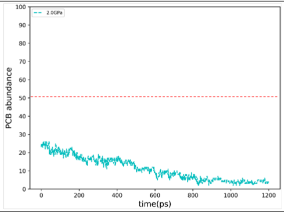	92
**DCT**	1800	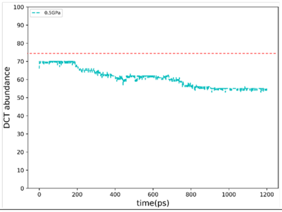	25	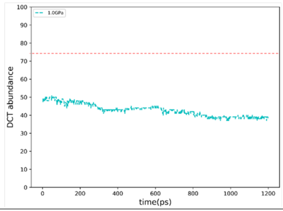	48	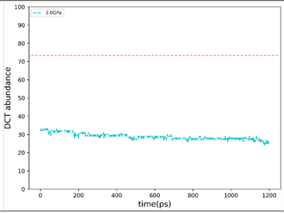	64
	2100	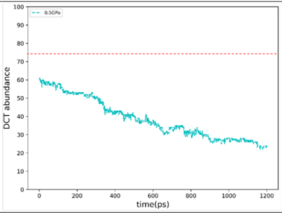	68	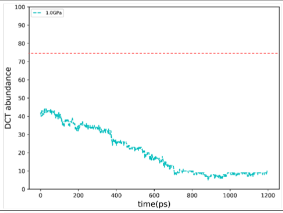	86	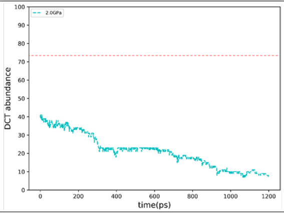	89
**DCB**	1800	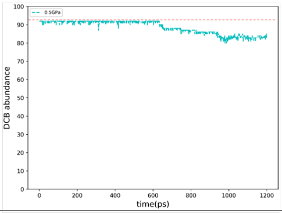	10	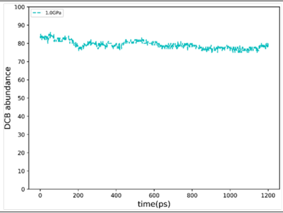	13	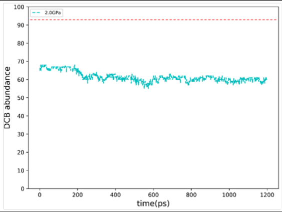	35
	2100	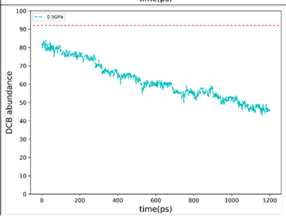	50	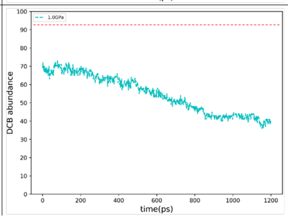	57	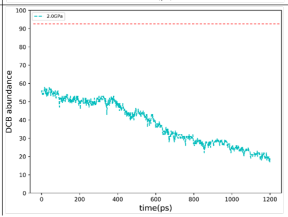	79

Figure S3: Time development of lost molecules (PCB 77, DCT, and DCB) due to thermal loading (1800 K and 2100 K) and pressure (0.5 GPa, 1.0 GPa, 2.0 GPa). Note that in an effort to keep the simulation box volume constant, the number of molecules is different for the molecular types (50, 73, and 92, respectively, indicated by the dashed red line).

T (K)	Shear rate	0.5 GPa	molecules lost	1.0 GPa	molecules lost	2.0 GPa	molecules lost
1800	-	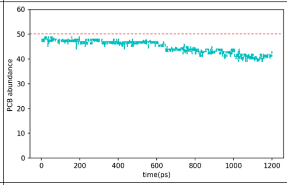	18%	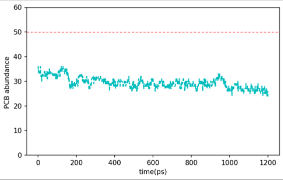	50%	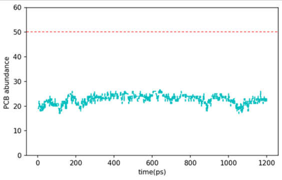	56%
1800	1E+10	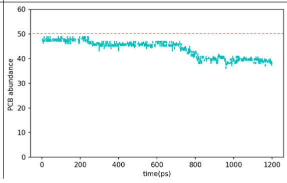	24%	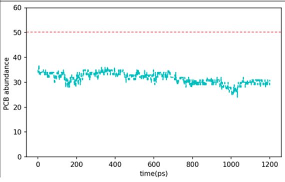	38%	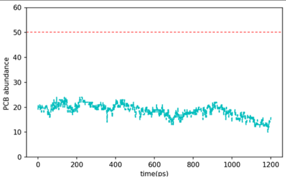	68%

Figure S4: Influence of the shear rate on the decomposition of PCB 77 at 1800 K for three different normal pressures. Top row: no shear, bottom row: shear rate 10^{10} 1/s.

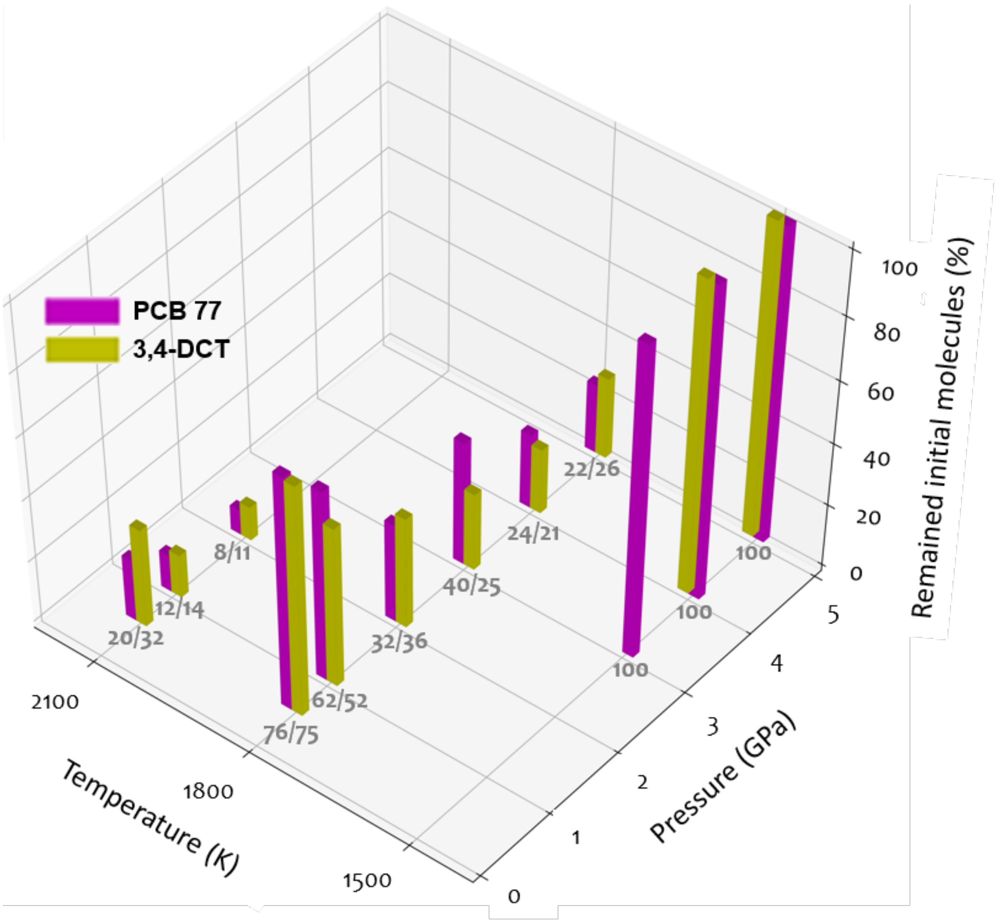

Figure S5: On the similarity of the decomposition of PCB 77 and DCT: percentages of the remaining initial molecules as functions of temperature and pressure.

	1800 K				2100 K			
	Cl-reactivity map	No. of reactions	Chem. types	remaining PCB 77 (%)	Cl-reactivity map	No. of reactions	Chem. types	remaining PCB 77 (%)
**0.5 GPa**	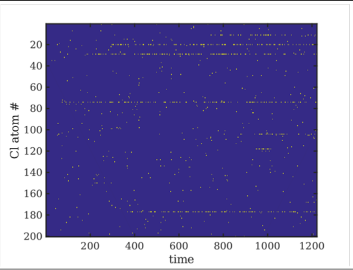	994	14	80%	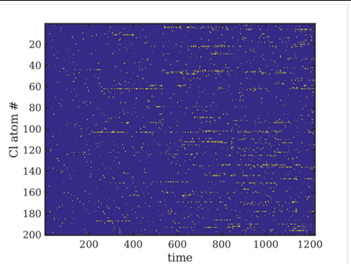	2914	85	44%
**1.0 GPa**	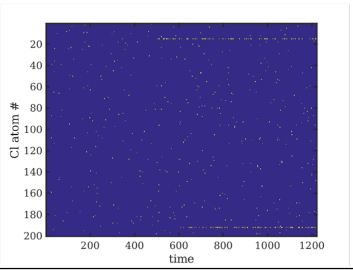	600	10	92%	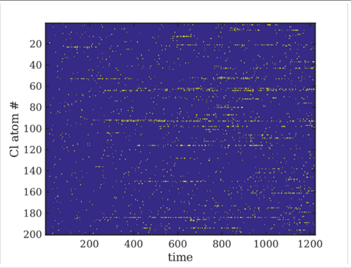	3089	76	36%
**2.0 GPa**	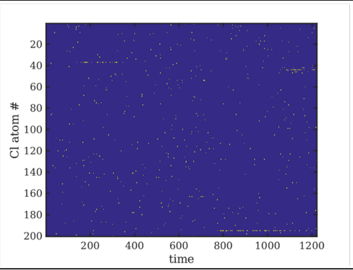	615	14	84%	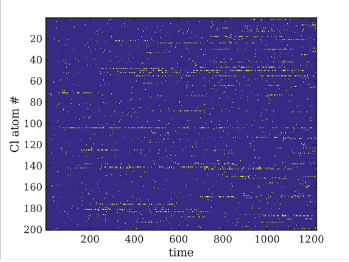	3389	130	32%

Figure S6: Reactivity maps for all Cl atoms in PCB 77 systems subjected to temperatures of 1800 K and 2100 K as well as normal pressures of 0.5 GPa, 1.0 GPa, and 2.0 GPa. Yellow dots denote a change in the bonding state of that atom.

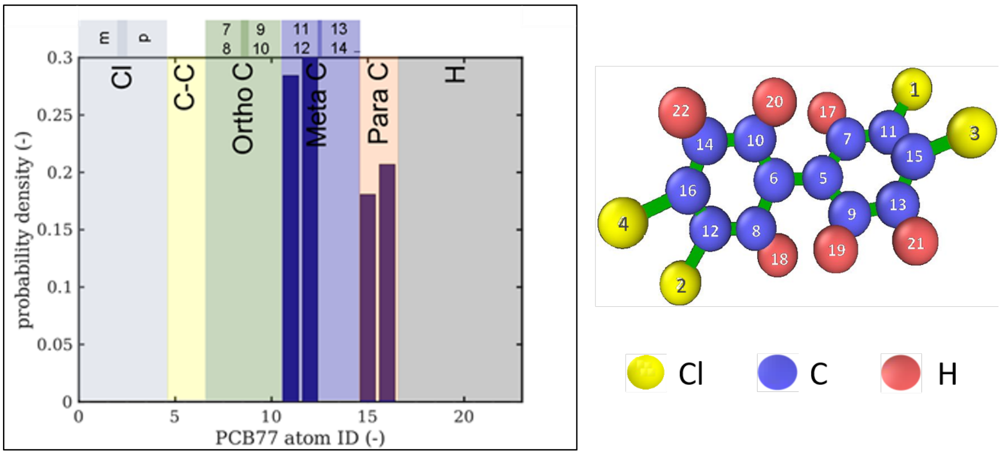

Figure S7: Probability density for Cl being bonded to the meta and para positions.

Table S1: Mean absolute percentage deviation (%) relative to PCB 77 property values. Closer similarity to PCB 77 is highlighted in green.

Property	DCT	DCB
Dipole moment	35.15	10.28
HOMO-LUMO gap	18.51	21.79
Isotropic Polarizability	47.03	53.85
Anisotropic Polarizability	59.96	66.31
Isotropic Magnetizability	41.40	47.99
Diamagnetic contribution:	77.65	83.73
Paramagnetic contribution:	78.66	84.72
1st anisotropy:	42.28	38.60
2nd anisotropy:	27.69	30.82
Isotropic g-tensor	10.20	38.12
Ionisation Potential	5.35	9.19
Fe-Cl BDE	0.52	0.32
C-Cl BDE	0.06	0.32
C-Cl Bond length	0.13	0.09
Fe-Cl Bond length	0.12	0.07
Electronegativity	9.52	6.00
Chemical hardness	18.51	21.79
Electrophilicity index	55.21	57.80
Electrophilic susceptibility	67.83	75.98
Hyper-hardness	25.02	31.09
Total Average	31.04	33.94
